# Progeria and Aging—Omics Based Comparative Analysis

**DOI:** 10.3390/biomedicines10102440

**Published:** 2022-09-29

**Authors:** Aylin Caliskan, Samantha A. W. Crouch, Sara Giddins, Thomas Dandekar, Seema Dangwal

**Affiliations:** 1 Department of Bioinformatics, Biocenter, University of Würzburg, 97074 Würzburg, Germany; 2 Stanford Cardiovascular Institute, Department of Medicine, Stanford University School of Medicine, Stanford, CA 94305, USA

**Keywords:** progeria, aging, omics, RNA sequencing, bioinformatics, sun exposure, HGPS, IGFBP2, ACKR4, WNT

## Abstract

Since ancient times aging has also been regarded as a disease, and humankind has always strived to extend the natural lifespan. Analyzing the genes involved in aging and disease allows for finding important indicators and biological markers for pathologies and possible therapeutic targets. An example of the use of omics technologies is the research regarding aging and the rare and fatal premature aging syndrome progeria (Hutchinson-Gilford progeria syndrome, HGPS). In our study, we focused on the in silico analysis of differentially expressed genes (DEGs) in progeria and aging, using a publicly available RNA-Seq dataset (GEO dataset GSE113957) and a variety of bioinformatics tools. Despite the GSE113957 RNA-Seq dataset being well-known and frequently analyzed, the RNA-Seq data shared by Fleischer et al. is far from exhausted and reusing and repurposing the data still reveals new insights. By analyzing the literature citing the use of the dataset and subsequently conducting a comparative analysis comparing the RNA-Seq data analyses of different subsets of the dataset (healthy children, nonagenarians and progeria patients), we identified several genes involved in both natural aging and progeria (KRT8, KRT18, ACKR4, CCL2, UCP2, ADAMTS15, ACTN4P1, WNT16, IGFBP2). Further analyzing these genes and the pathways involved indicated their possible roles in aging, suggesting the need for further in vitro and in vivo research. In this paper, we (1) compare “normal aging” (nonagenarians vs. healthy children) and progeria (HGPS patients vs. healthy children), (2) enlist genes possibly involved in both the natural aging process and progeria, including the first mention of IGFBP2 in progeria, (3) predict miRNAs and interactomes for WNT16 (hsa-mir-181a-5p), UCP2 (hsa-mir-26a-5p and hsa-mir-124-3p), and IGFBP2 (hsa-mir-124-3p, hsa-mir-126-3p, and hsa-mir-27b-3p), (4) demonstrate the compatibility of well-established R packages for RNA-Seq analysis for researchers interested but not yet familiar with this kind of analysis, and (5) present comparative proteomics analyses to show an association between our RNA-Seq data analyses and corresponding changes in protein expression.

## 1. Introduction

Aging is characterized as a time-dependent functional decline leading to progressive loss of physiological functions and deterioration [1]. It is known as the primary risk factor for several major pathologies, including cardiovascular disorders, diabetes, neurodegenerative diseases, and cancer [1].

López-Otín et al. (2013) have defined the hallmarks of aging, aiming for a similar effect on aging research as Hanahan and Weinberg’s hallmarks of cancer had on cancer research by contributing to the momentum cancer research has gained in recent decades [1]. While the hallmarks of aging offer an excellent overview of biogerontology, Gems and de Magalhães (2021) pointed out the need for more research to understand the processes involved in aging [2]. At the moment, the “hallmarks” do not completely capture the complex process of aging [2]. In addition, not all mechanisms observed in aging are exclusive to aging, such as dysregulation of the epigenome is also observed independent of age in overt disease [3]. Furthermore, the processes involved in aging are interconnected, as Kennedy et al. (2014) elaborate in their publication describing the seven pillars of aging [4]. Therefore, there is, of course, more to aging than a single explanatory paradigm can describe [2]. Hence, it is vital to collect and analyze data, for instance, by comparing protein interactions and pathways. Meta-analysis studies like ours and omics analyses in general can reveal important age-affected pathways, especially when comparing different age groups or different conditions, such as comparing nonagenarians with healthy young persons as well as progeria patients. As for all in silico analyses, subsequent in vitro and in vivo analyses are necessary before the results can be brought to clinical practice. However, the combination of computational methods will result in more supporting and clarifying efforts such as the seven pillars of aging or the hallmarks of aging.

The technical progress in research, which becomes obvious by looking at disciplines such as “-omics”, also contributes to the growing knowledge and understanding of the processes involved in aging. In general, the ending “-omics” indicates a global or comprehensive assessment of a kind of molecule [5].

Like the first “omics” discipline, genomics, which focuses on entire genomes instead of solely studying single genes [5], all kinds of “-omics” focus on a global or comprehensive assessment of a certain kind of molecule [5]. Transcriptomics, for instance, analyzes RNA levels in a qualitative and quantitative manner [5]. By comparing high-throughput sequencing data of healthy individuals and individuals affected by a disease, it is possible to gain a better understanding of various human pathologies including aging-related diseases.

An example of the progress in aging research is the rare and fatal premature aging syndrome progeria (Hutchinson-Gilford progeria syndrome, HGPS). The disease was first described in 1886 by Hutchinson [6] and in 1897 by Gilford [7], and was brought to attention as a detailed case study in 1913 [7], emphasizing the need for further knowledge and research [7,8].

Progeria is often diagnosed relatively early in childhood, and as the name suggests, several of the symptoms of progeria also occur with old age, including hair graying or loss, skin thinning, and osteopenia/osteoporosis [9] as well as diminished joint mobility [10]. Besides aging symptoms, progeria patients often suffer from cardiovascular problems [11] as well as stroke [11], which result in premature death and an average life expectancy of about 13 years.

In 2003, 90 years after progeria was ardently brought to attention, a mutation in LMNA (Lamin A/C) was identified as the cause of HGPS by the Progeria Research Foundation’s collaborative research team [12]. Due to a dominant 1824C > T mutation, which was found to be the predominant cause of progeria, a cryptic splice donor site gets activated [13,14]. This is causing the formation of a truncated prelamin A, named progerin, which is missing 50 amino acids due to internal deletion and is not processed into normal lamin A [13,14].

Thanks to the efforts of the Progeria Research Foundation (PRF) and the worldwide participation of progeria patients and their families, less than two decades later, two successful clinical trials aiming to treat HGPS and increase the life expectancy of HGPS patients have been performed [15,16] and a third trial is expected to be completed in 2023 (NCT02579044). Additionally, the first medication against progeria has been FDA (U.S. Food and Drug Administration) approved [17] and is in the process of being approved in Europe [18]. Besides searching for a cure, the PRF also supports progeria and aging research by other scientists, for instance, by sharing fibroblasts that were donated by HGPS patients [19].

Using human fibroblasts, Fleischer et al. (2018) generated a comprehensive set of genome-wide RNA sequencing (RNA-Seq) profiles to develop a computational method to predict the biological age [19]. The dataset contains RNA-Seq data of dermal fibroblasts donated by ten progeria patients and 133 “apparently healthy” individuals (aged 1 to 96 years according to their metadata) [19]. Their dataset is publicly available on the Gene Expression Omnibus (GEO) database [20] under accession number GSE113957 [19]. Since its publication in November 2018, the article by Fleischer et al. has been cited 37 times in PubMed (until May 2022), with 13 of the citing papers mentioning the use of the GSE113957 dataset from the Fleischer et al. paper [21,22,23,24,25,26,27,28,29,30,31,32,33] (as described in Table 1). According to the publications in PubMed, the dataset alone contributed to 8 studies investigating aging [21,22,23,24,25,26,27,28].

The frequent use of the dataset indicates the broad use and the value of the dataset for research, not only in aging research but also in bioinformatics in general. Keeping in mind that there might be further studies either still in preparation or not indexed in PubMed, the possible applications of the dataset are nowhere near exhausted.

Although several of the publications citing the use of the GSE113957 dataset by Fleischer et al. focused on aging, they investigated different aspects and effects. Therefore, to demonstrate the variety of research and highlight the value addition in the current study, we summarize this information in Table 1. Additionally, as shown in Table 1, we provide the first comparative study looking both at aging (nonagenarians vs. healthy children) and progeria (HGPS patients vs. healthy children). The other studies focused either on aging alone (comparing healthy children to people of (extreme) age) or progeria alone (comparing healthy children and progeria patients).

Besides the extensive comparison data for creating and testing computational analysis methods, the RNA-Seq data can be combined with new analysis methods and the growing knowledge regarding pathways and protein interactions. This will enable further insights and lead to new findings regarding fibroblasts, aging, pathways, and potential relationships and interactions.

In the present study, we used the RNA sequencing data to demonstrate the power of bioinformatics to reveal important differences between normal aging, progeria, and young fibroblasts in terms of pathways, proteins, and protein networks. We identified several genes potentially involved both in natural aging and progeria (KRT8, KRT18, ACKR4, CCL2, UCP2, ADAMTS15, ACTN4P1, WNT16, IGFBP2). Further genes and pathways analysis confirmed their roles in aging, suggesting the need for further in vitro and in vivo research. We considered three subgroups of the dataset: healthy children, nonagenarians, and HGPS patients. As HGPS patients suffer from many conditions associated with old age, we were interested in the differences and similarities between HGPS patients and nonagenarians, as well as between healthy children and children suffering from HGPS. Additionally, we compared the RNA-Seq data of healthy children and nonagenarians to see the differences in gene expression occurring during natural aging. While this study is the first extensive multi-omics comparative study comparing normal aging based on the dataset by Fleischer et al. [19] with progeria, to reveal the above genes and detailed further differences in genes and pathways for normal and pathological aging, only further analysis has to find out, how far the changes in pathways and gene expression found in nonagenarians found here are only markers or really makers of successful aging.

All tools used for this analysis are freely available R packages or software. To encourage such analyses for other pathophysiological conditions and stimulate transcriptome analysis, we will give some details on the different tools and where to find vignettes and workflows explaining the use of the respective tools (Supplementary Materials, Document S1).

## 2. Materials and Methods

In this study, already published, publicly available data is analyzed. Thus, ethical approval and patient consent were not necessary.

### 2.1. Hardware and Software

All analyzes were performed on a PC with AMD Ryzen 9 3900X, 12-Core Processor, 64.0 GB RAM, 64-Bit-Operating System, and an x64-based processor. A virtual Ubuntu environment (Ubuntu 20.04.2 LTS (OS-Type: 64-bit) running on a virtual machine (Virtual Box 6.1.34)) was used for data download, quality control, and alignment. The subsequent data analysis was performed using RStudio (2022.02.0+443 “Prairie Trillium” Release (9f7969398b90468440a501cf065295d9050bb776, 2022-02-16) for Ubuntu) with R version 4.2.0 (2022-04-22) [34]. Cytoscape analyses were performed using Cytoscape for Windows (64-bit, version 3.9.1, on Windows 10).

### 2.2. RNA-Seq Data

The single-end stranded RNA-Seq data of the GEO [35] dataset GSE113957 was downloaded via NCBI’s SRA Run Selector and checked for quality using FastQC (version 0.11.9) [36] and MultiQC (version 1.12) [37]. The dataset was generated by Fleischer et al. (2018) [19]. It contains RNA-Seq data of human fibroblast cell lines derived from 10 progeria patients (Hutchinson-Gilford progeria syndrome (HGPS)) and 133 fibroblast cell lines derived from “apparently healthy” individuals [19]. According to the metadata provided via NCBI’s SRA Run Selector, the healthy individuals were aged between 1 and 96.

For this study, only the samples of the progeria patients aged 6 to 8 years (5 samples), the samples of healthy children in the same age group (age 6 to 9, 6 samples), and the samples of the individuals aged 90+ (7 samples) were analyzed.

### 2.3. Data Preprocessing

The RNA-seq data was aligned to GENCODE v39 [38] using the standard protocols for STAR (version 2.7.10a) [39] and RSEM (version 1.3.1) [40]. After STAR alignment, the transcripts were subsequently quantified with RSEM.

### 2.4. Identification of DEGs

We performed DESeq2 (version 1.36.0, with apeglm version 1.18.0, using tximport version 1.24.0 for importing the data in R) [41,42,43] analyses to find differences between the two groups: (1) HGPS patients vs. healthy children and (2) 90-year-olds vs. healthy children. Differentially expressed genes (DEGs) with a *p*-value < 0.05 were considered significant. The log2 fold change threshold values were set to > 1 for upregulated genes and < −1 for downregulated genes.

### 2.5. Data Visualization

Principal component analysis (PCA) was performed using the DESeq2 package, and gene expression and DEGs were visualized in the form of volcano plots (EnhancedVolcano, version 1.14.0) [44] and heatmaps (pheatmap version 1.0.12) [45].

Additionally, gene expression was visualized using IsoformSwitchAnalyzeR (version 1.18.0) [46], which supports data from various quantification tools, including RSEM [46]. To calculate gene expression, IsoformSwitchAnalyzeR can take count and abundance values into account and calculates gene expression by adding up the abundance values of all isoforms related to the respective gene [46]. The gene expression function of the IsoformSwitchAnalyzeR package was used for three different comparisons: (1) HGPS patients vs. healthy children, (2) 90-year-olds vs. healthy children, and (3) HGPS patients vs. 90-year-olds.

### 2.6. Pathway Enrichment Analysis

Databases such as the Molecular Signatures Database [47,48,49,50], provide annotated gene sets that can be used for further analyses, including hallmark gene sets and ontology gene sets. The hallmarks gene set can be envisioned as a starting point for further analyses [48]. Biological ontologies, such as Gene Ontology (GO) [49], provide knowledge about genes and their functions [51,52]. The gene ontology offers information on the sub ontologies that represent protein function: biological process (BP), cellular component (CC), and molecular function (MF) [53].

The enrichment analyses of the hallmark gene set and the GO BPs gene set were calculated using MSigDB (version 7.5.1) [47,48,49,50] and visualized as bar plots, CNET plots, and heat plots using clusterProfiler (version 4.4.2) [51,54], enrichplot (version 1.16.1) [55], and ggplot2 (version 3.3.6) [56]. The heat plot function of the enrichplot package [55], which is also embedded in clusterProfiler [51,54], combines the functionalities of a heatmap and a CNET plot by displaying relationships—e.g., the genes involved in a specific pathway—as a heatmap [55].

### 2.7. Protein–Protein Interactions

The Search Tool for Retrieval of Interacting Genes/Proteins (STRING) database [57] and web tool is a meta-resource for analyzing protein–protein interactions [57,58]. It is based on analyzing the ‘functional association’ of proteins, which is described as a link between two proteins that both contribute to a biological function [57].

The significant DEGs of interest were mapped to STRING using the official gene symbol as input for the web app (https://string-db.org/, version 11.5, accessed on 22 July 2022) with a fullstringnetwork medium confidence of 0.4 and visualized via Cytoscape [59].

The open-source software project Cytoscape was developed as a modeling environment for the integration of molecular network interaction data. Its organizing metaphor is a network graph [59]. The nodes of the graph are molecular species that are connected via intermolecular interactions, which are represented as edges or links between the nodes. It supports various automated network layout algorithms and allows the user to visualize their data in the form of a network [59]. Furthermore, Cytoscape is designed to allow the implementation of additional plug-ins addressing biological problems [59].

We used Cytoscape [59] to further analyze and visualize the STRING database results for our genes of interest. Additionally, using Cytoscape [59], we visualized the log2fold changes of the DEGs that were calculated during DESeq2 analysis [41] (for aging and progeria) and the average log2fold changes of the common DEGs in both conditions (calculated by adding the respective values and subsequently dividing them by 2).

### 2.8. Venn Diagrams

Venn diagrams were introduced almost 150 years ago as a method of visually representing classes and elements contained in one or several of these classes using intersecting circles [60]. Venn Diagrams can represent results that are rather difficult to explain in words in an intuitively understandable graphic representation. Therefore, they can be used to visualize overlapping genes between several groups. Besides that, Venn diagram tools, like the web app Venny [61] (https://bioinfogp.cnb.csic.es/tools/venny/, accessed on 22 July 2022), also offer to extract lists of every section of the Venn diagram [61].

The different genes of interest for the respective groups were visualized using the online tool Venny (version 2.1.0) [61]. Depending on the comparison, two or three lists of DEGs or pathways were uploaded in Venny, which automatically visualized overlaps and offers the option to save the resulting figure and the elements contained in the overlaps.

### 2.9. miRNA Prediction

For predicting microRNA (miRNA) interactions, we used miRNet (https://www.mirnet.ca/, version 2.0, accessed on 22 July 2022), a web-based platform for miRNA analysis. The input data is integrated with prior knowledge, including miRNA-target interactions, transcription factors, and single nucleotide polymorphisms [62], and the results can be visualized as a network using Cytoscape [59]. This allows the prediction of miRNAs that might be regulated by genes of interest. We performed three miRNA predictions, using the DEGs involved in aging, progeria, and the common DEGs of both conditions as the respective input data.

### 2.10. NicheNet: Finding Ligand–Receptor Interactions Based on Prior Knowledge

Since the growing knowledge of biological processes such as gene interactions and cellular communication is a cornerstone in data analysis, Türei et al. (2021) generated Omnipath, a comprehensive database combining over a hundred different resources covering protein interaction, transcriptional and post-transcriptional regulation, and cellular signaling [63].

NicheNet is a computational method developed for combining the prior knowledge archived in databases such as Omnipath with gene expression data, enabling the user to analyze prioritized ligand–target interactions as well as intracellular signaling [64]. Although NicheNet offers its own database, it can also be combined with other databases as the source of the prior knowledge on which the subsequent NicheNet analysis is based.

In this study, we followed the workflow for combining NicheNet (version 1.1.0) [64] and Omnipath data (via OminpathR, version 3.4.0) [63] previously described by Türei et al. (2021) [63]. The workflow enables prediction of prioritized interaction partners for DEGs involved in a pathway of interest (via fgsea, version 1.22.0) [65], which can offer further insights in network analysis [63].

### 2.11. Figures and Additional Packages

While the graphical abstract was created using BioRender (https://biorender.com/, accessed on 22 July 2022), the figures containing analysis results were arranged using R/RStudio. The following helpful R packages were used for figure creation or as additional packages/dependencies of the packages used for analyses and figure creation: cowplot (version 1.1.1) [66], ggplotify (version 0.1.0) [67], magick (version 2.7.3) [68], scatterplot3d (version 0.3.41) [69], scales (version 1.2.0) [70], viridis (version 0.6.2) [71], plotly (version 4.10.0) [72], RcolorBrewer (version 1.1.3) [73], ggupset (version 0.3.0) [74], ggnewscale (version 0.4.7) [75], pathview (version 1.36.0) [76], ggridges (version 0.5.3) [77], europepmc (version 0.4.1) [78], BiocManager (version 1.30.18) [79], org.Hs.eg.db (version 3.15.0) [80], tidyverse (version 1.3.1) [81], dplyr (version 1.0.9) [82].

## 3. Results

HGPS patients suffer from old age symptoms, therefore, we were interested in the differences and similarities of natural, chronological aging, as seen in individuals of extreme age like nonagenarians, and premature or accelerated aging, as it can be observed in progeria patients. For our study, we compared three subsets of the RNA sequencing data within Fleischer et al.’s publicly available GEO dataset GSE113957 [19]: HGPS patients, nonagenarians (90s, aged 90 to 96 according to the metadata submitted with the GEO dataset), and healthy children (Supplementary Materials, Table S1). To find genes related to aging and aging-related pathologies, we performed DESeq2 analyses comparing healthy children with progeria patients and nonagenarians, respectively. The results of both analyses were compared, focusing on DEGs, GO enrichment/pathways, microRNAs (miRNAs, miRs), and interaction partners.

Since Gordon et al. (1993–2022) reported that death due to complications of HGPS such as cardiac or cerebrovascular disease most often occurs in the age range between six and 20 years [83], we decided to focus on children suffering from HGPS aged six or older (HGPS, ages 6 to 8 years). RNA sequencing data samples of healthy children of the same age group (Healthy Kids, aged 6 to 9) were used as controls.

### 3.1. Differences and Similarities between Old Age and HGPS

**Figure 1:**
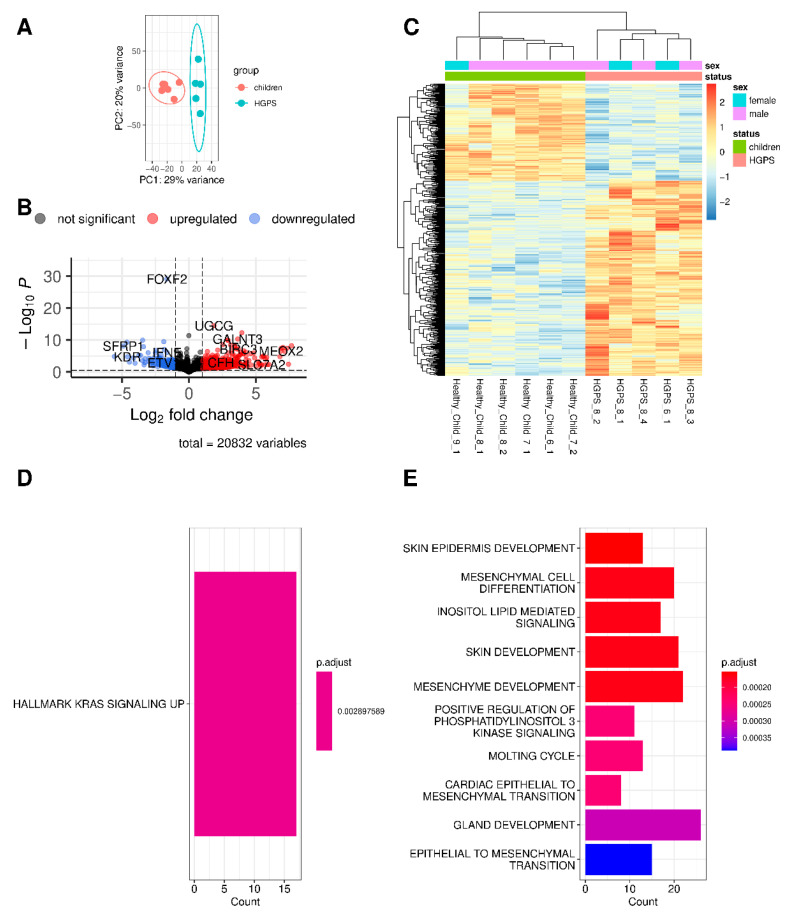
Comparing gene expression in RNA sequencing data of HGPS patients and healthy children (progeria/accelerated aging). (**A**) Principal Component Analysis (PCA): HGPS patients (blue dots) compared to healthy children (controls, red dots). (**B**) Volcano plot visualizing differentially expressed genes (DEGs): significantly upregulated genes are shown as red dots, significantly downregulated genes as blue dots, gray dots symbolize genes without significant changes in gene expression. (**C**) Heatmap and hierarchical clustering of the DEGs. (**D**) Bar plot of enriched hallmark pathways. (**E**) Bar plot of GO enriched biological processes.

**Figure 2:**
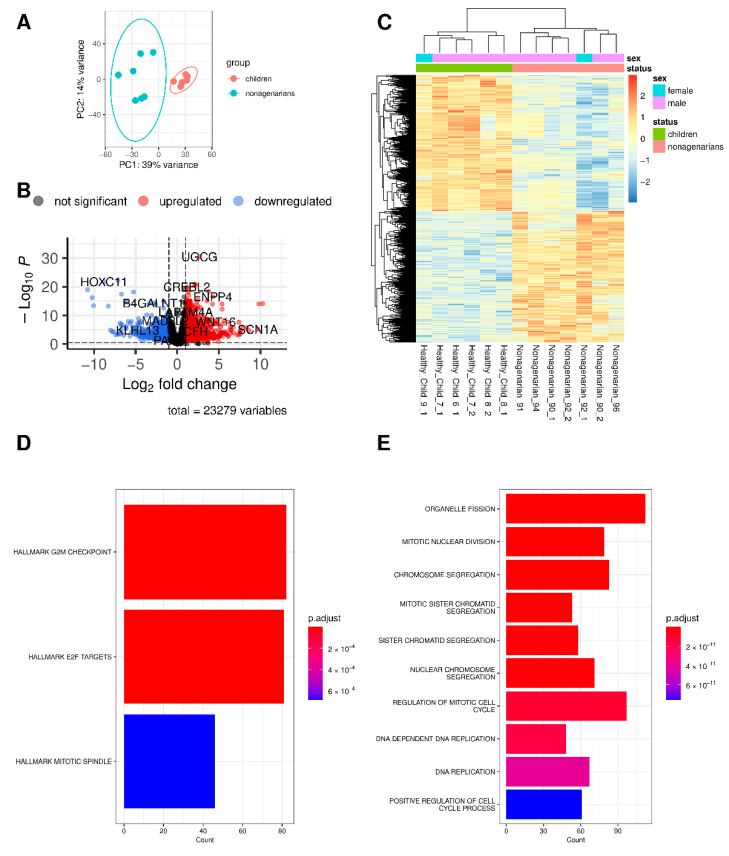
Comparing gene expression in RNA sequencing data of nonagenarians and healthy children (normal/chronological aging). (**A**) Principal Component Analysis (PCA): nonagenarians (blue dots) compared to healthy children (controls, red dots). (**B**) Volcano plot visualizing differentially expressed genes (DEGs): significantly upregulated genes are shown as red dots, significantly downregulated genes as blue dots, gray dots symbolize genes without significant changes in gene expression. (**C**) Heatmap and hierarchical clustering of the DEGs. (**D**) Bar plot of enriched hallmark pathwayhas. (**E**) Bar plot of GO enriched biological processes.

The gene expression of HGPS patients (Figure 1) and nonagenarians (Figure 2) were compared with the gene expression of healthy children using DESeq2 analysis [41].

Principal Component Analysis (PCA, Figure 1A and Figure 2A) indicates differences in gene expression between HGPS patients and healthy children (progeria, premature or accelerated aging) and nonagenarians and healthy children (aging), respectively. DEGs are visualized as volcano plot [44] (Figure 1B and Figure 2B) and as heatmap [45] with hierarchical clustering (Figure 1C and Figure 2C). Upregulation is visualized in red, downregulation in blue. Comparing HGPS patients and healthy children (HGPS vs. healthy children, Figure 1) resulted in 497 DEGs, with 332 genes being upregulated and 165 downregulated in progeria. In natural aging (90s vs. healthy children, Figure 2), 2743 genes are differentially expressed, with 1350 DEGs being upregulated and 1393 being downregulated (Supplementary Materials, Tables S2, S5, and S6).

Hallmark enrichment analysis and Gene Ontology enrichment analysis for biological processes (BPs) were conducted using clusterProfiler [51,54] and the respective gene sets available via the Molecular Signatures Database (MsigDB) [47,48,49,50]. While normal aging (Figure 2D,E, Supplementary Materials Figures S3 and S4) appears to affect the cell cycle G2/M checkpoint (G2M checkpoint), E2F targets, and the mitotic spindle assembly (hallmark MITOTIC_SPINDLE), progeria is only associated with KRAS signaling up, the genes upregulated by KRAS (Kristen rat sarcoma virus) activation (Figure 1D,E, Supplementary Materials Figures S1 and S2).

GO enrichment analysis for BPs using clusterProfiler [51,54] with the respective DEGs indicates which BP pathways might be affected by the differences in gene expression. In accelerated aging, 171 BP pathways were significantly enriched. The top ten enriched pathways of the clusterProfiler analysis are visualized as bar plots in Figure 1E. Here, pathways related to skin and skin development are among the top enriched pathways. Among the 189 significantly enriched BP pathways found in natural aging, several pathways related to the cell cycle were among the top ten enriched pathways (Figure 2E). The top three pathways of both comparisons and their related DEGs are visualized as CNET plots in the Supplementary Materials (progeria in Figure S2, aging in Figure S4), demonstrating that these processes are also interconnected via the involved DEGs.

### 3.2. Changes in Gene Expression in Progeria and Normal Aging

**Figure 3:**
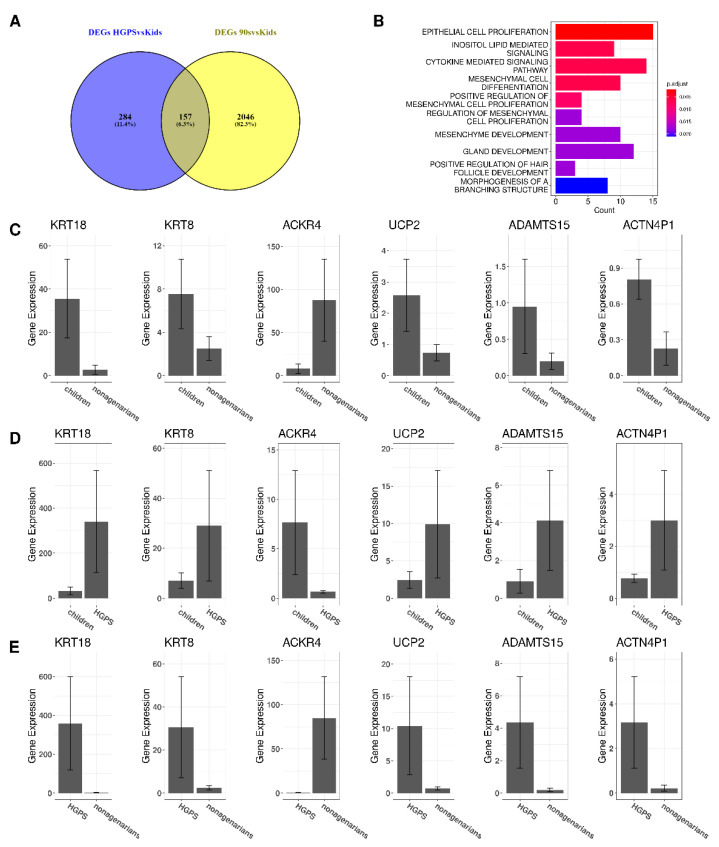
Comparison of accelerated and natural aging. (**A**) Common DEGs in accelerated aging (DEGs between HGPS patients and healthy children, blue) and normal aging (DEGs between nonagenarians and healthy children, yellow) are visualized as Venn diagram. (**B**) The ten most enriched biological processes with GO enrichment using the common DEGs of aging and HGPS (overlap in A). (**C**) Gene expression of the six genes regulated in opposite directions, differences in gene expression between children and nonagenarians. (**D**) Gene expression of the six genes regulated in opposite directions, differences in gene expression between children and progeria patihass. (**E**) Gene expression of the six genes regulated in opposite directions, differences in gene expression between progeria patients and nonagenarians.

Accelerated and natural aging share changes in gene expression. The Venn diagram in Figure 3A shows that both comparisons have 157 DEGs in common. However, not all of these DEGs are regulated in the same direction in both comparisons. The differences in gene expression of the six differently regulated genes (Figure 3C–E) are visualized using the R-package IsoformSwitchAnalyzeR [46].

Comparing the changes in gene expression (log2foldchanges) of the 157 DEGs revealed that six DEGs (KRT18 (Keratin 18), KRT8 (Keratin 8), ACKR4 (atypical chemokine receptor 4), UCP2 (Uncoupling Protein 2), ADAMTS15 (ADAM metallopeptidase with thrombospondin type 1 motif 15), and ACTN4P1 (Actinin alpha 4 pseudogene 1)) were regulated in opposite directions. For instance, while HGPS patients express more KRT18 than healthy children (Figure 3D), the expression of KRT18 appears to be reduced upon normal aging as nonagenarians express less KRT18 than both healthy children (Figure 3C) and children affected with HGPS (Figure 3E).

Performing GO enrichment with the 157 DEGs that both types of aging have in common results in 27 biological processes. The top ten enriched biological processes of the clusterProfiler analysis using the common DEGs are visualized in Figure 3B. Considering the common DEGs, Epithelial Cell Proliferation (ECP) appears to be the most enriched pathway. Additionally, ECP is also enriched in both comparisons, although it is not among the top ten enriched BPs in accelerated and normal aging.

**Figure 4:**
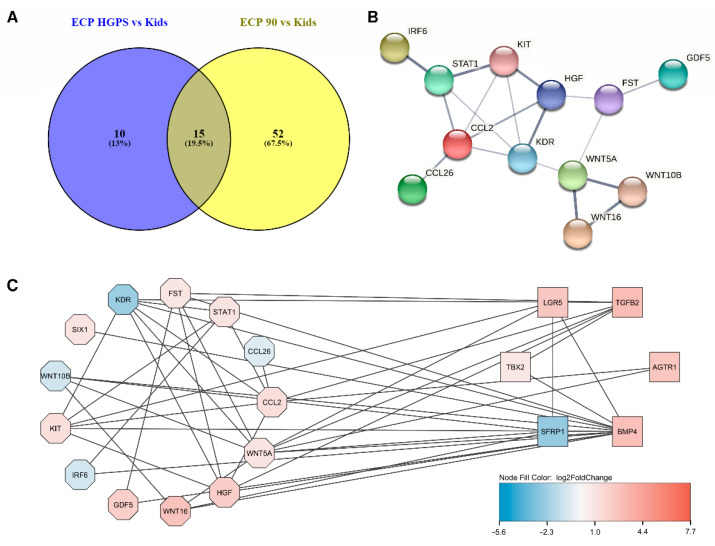
Comparison of accelerated and natural aging. (**A**) The visualization of the DEGs involved in Epithelial Cell Proliferation (ECP) differentially expressed in HGPS (blue) and old age (yellow) shows that both have 15 DEGs in common. (**B**) The common DEGs of old age and HGPS involved in ECP are visualized as STRING network (applying fullstringnetwork medium confidence of 0.4). (**C**) Visualization of the DEGs involved in ECP (by using STRING database and Cytoscape): common DEGs (octagons) and DEGs specific for HGPS (rectangles) and the respective log2foldchanges (blue downregulated, red upregulated).

Comparing the DEGs in HGPS that are involved in ECP with the DEGs in old age and ECP shows that both groups have 15 genes of the ECP pathway in common: WNT16 (Wnt Family Member 16), CCL26 (C-C Motif Chemokine Ligand 26), HGF (Hepatocyte Growth Factor), PTPRN (Protein Tyrosine Phosphatase Receptor Type N), CCL2 (C-C Motif Chemokine Ligand 2), WNT5A (Wnt Family Member 5A), STAT1 (Signal Transducer And Activator Of Transcription 1), IRF6 (Interferon Regulatory Factor 6), GDF5 (Growth Differentiation Factor 5), SIX1 (SIX Homeobox 1/Sine Oculis Homeobox Homolog 1), KDR (Kinase Insert Domain Receptor), FST (Follistatin), KIT (KIT Proto-Oncogene, Receptor Tyrosine Kinase), NKX3-1 (NK3 Homeobox 1), and WNT10B (Wnt Family Member 10B) (Figure 4A). Analyzing these genes in the STRING database [58] (Figure 4B) shows that almost all of these DEGs are linked with each other.

Further analysis in Cytoscape [59] was performed by combining the STRING results and the changes in gene expression that were evaluated. Cytoscape allows visualization of the ECP-related DEGs. Figure 4C visualizes the ECP-related DEGs both conditions have in common with ECP (octagons) and the ECP-related DEGs specific for comparing HGPS patients and healthy children (rectangles). The log2foldchanges derived from the DESeq2 analysis are indicated by color, with blue symbolizing downregulation and red upregulation. The same analysis was performed by comparing the ECP-related common DEGs of both conditions and the DEGs that are related to ECP but only differentially expressed between nonagenarians and healthy children (Supplementary Materials, Figure S5).

### 3.3. The Different Pathways Involved in Progeria, Aging, and Both Conditions

**Figure 5:**
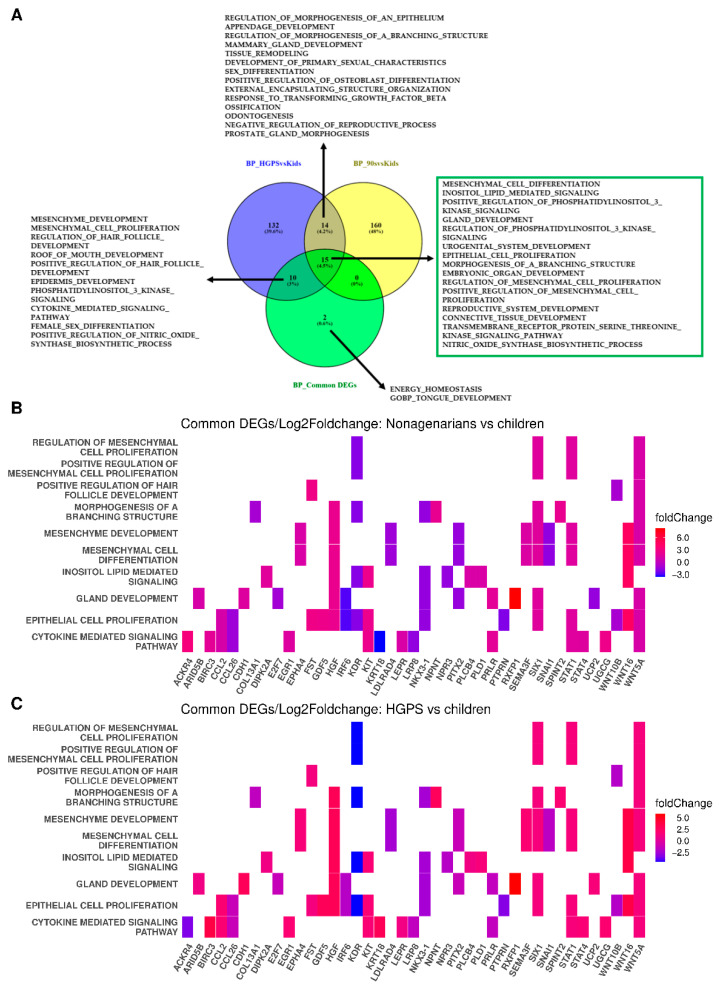
Differences in gene expression between HGPS and aging. (**A**) Venn diagram visualizing the GO BPs enriched in HGPS (blue), aging (yellow), and in the 157 common DEGs of progeria and aging. 15 BPs are enriched in all three analyses. (**B**) Heat plot visualizing the changes in gene expression in the DEGs involved in the 15 BPs using the changes in gene expression observed while comparing nonagenarians and healthy children (log2foldchanges of DESeq2 analysis). (**C**) Heat plot visualizing the changes in gene expression in the DEGs involved in the 15 BPs using the changes in gene expression observed while comparing progeria patients and healthy children (log2foldchanges of DESeq2 analysis). The differences in gene expression are indicated by color (red for upregulated, blue for downregulated). Notably, UCP2, which is involved in gland development, is upregulated in progeria but downregulated in aging. ACKR4, which is involved in the cytokine-mediated signaling pathway, is upregulated in aging (comparison of healthy children and nonagenarians) but downregulated in progeria (comparison of healthy children and progeria patients).

The Venn diagram in Figure 5A visualizes the BPs which were calculated for progeria (Figure 5A, blue circle), aging (Figure 5A, yellow circle), and the DEGs both conditions have in common (Figure 5A, green circle). The complete list of the respective BPs is available in the Supplementary Materials (Table S3).

To compare the changes in gene expression between progeria and aging, we analyzed the 15 common pathways as heat plots (Figure 5B,C). The *y*-axes show the 15 common pathways, while the genes involved in the respective pathways are indicated on the *x*-axes. The changes in gene expression were derived from the log2fold changes in gene expression in aging (Figure 5B) and progeria (Figure 5C), respectively.

Many genes show similar gene expression patterns and only differ in the log2foldchanges in gene expression. However, some genes are upregulated in one of the comparisons and downregulated in the other (see Figure 3C–E). One of these genes, UCP2, is also involved in gland development. While UCP2 is upregulated in progeria, it is downregulated in nonagenarians. ACKR4, which is also regulated in different directions, is involved in the cytokine-mediated signaling pathway.

**Figure 6:**
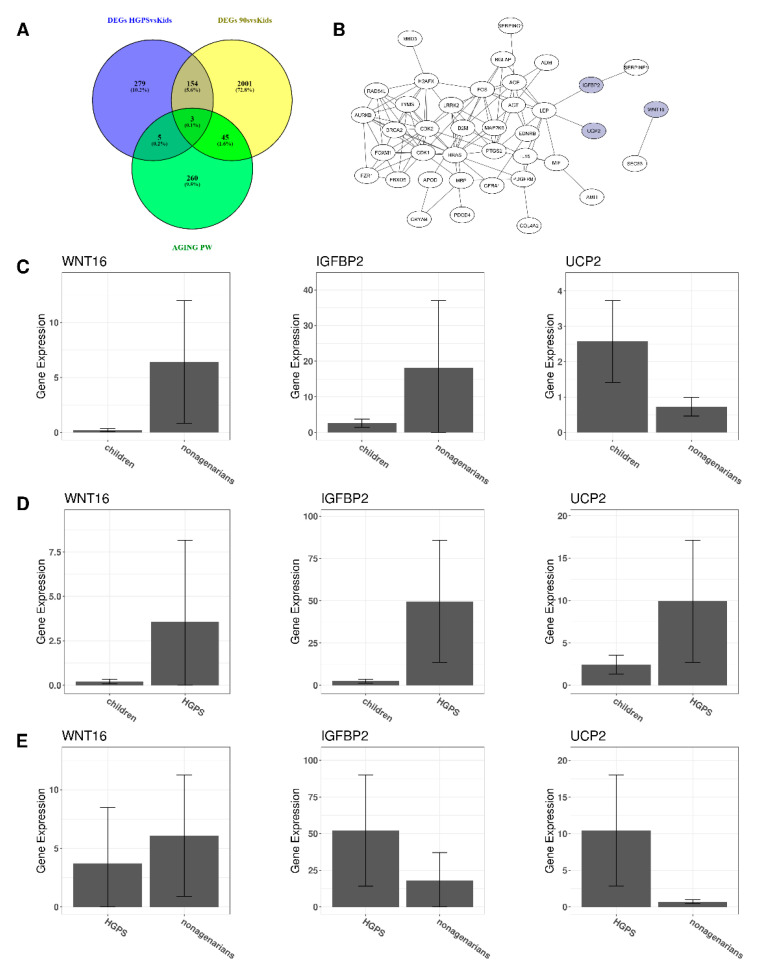
Network visualization of miRNAs related to WNT16, IGFBP2, and UCP2. (**A**) Comparing the genes involved in the Aging Pathway (green) with the DEGs in progeria comparison (blue) and the aging comparison (yellow) results in three common DEGs WNT16, IGFBP2, and UCP2. (**B**) Network visualization of the DEGs involved in the aging pathway and differentially expressed between healthy children and nonagenarians. The three DEGs that are also differentially expressed when comparing progeria patients and nonagenarians are highlighted in purple. (**C**) Gene expression of the three DEGs WNT16, IGFBP2, and UCP2, when comparing children and nonagenarians. (**D**) Gene expression of the three DEGs WNT16, IGFBP2, and UCP2, when comparing children and HGPS phasents. (**E**) Gene expression of the three DEGs WNT16, IGFBP2, and UCP2, when comparing progeria patients and nonagenarians.

The aging pathway was among the enriched pathways in aging but not in progeria. As we were especially interested in aging, we compared the DEGs of HGPS and aging with the genes known to be involved in the biological process “GOBP_AGING” (Figure 6A), which can be found in the Molecular Signatures Database (MSigDB) [48]. The DEGs between healthy children and nonagenarians also related to the aging pathway are shown in Figure 6B. The three DEGs that appear to be associated with the aging pathway, progeria and normal aging are highlighted in purple.

Besides UCP2, only WNT16 and IGFBP2 (Insulin Like Growth Factor Binding Protein 2) are DEGs in both conditions and are known to be involved in the aging pathway. Figure 6C–E visualizes the gene expression of the respective genes. Expression of WNT16 is higher in nonagenarians (Figure 6C) and progeria patients (Figure 6D) compared to healthy children. Comparing nonagenarians to progeria patients shows that nonagenarians have a slightly higher expression of WNT16 than HGPS patients (Figure 6E).

Nonagenarians express higher levels of IGFBP2 than healthy children (Figure 6C). Progeria patients present a higher IGFBP2 expression than healthy children (Figure 6D) and even higher IGFBP2 levels than nonagenarians (Figure 6E). Healthy children (Figure 6C) and progeria patients (Figure 6E) express more UCP2 than nonagenarians. At the same time, progeria patients have higher UCP2 levels than healthy children (Figure 6D).

### 3.4. Prediction of microRNAs and Visual Exploration of Interaction Partners of WNT16, IGFBP2, and UCP2

MicroRNAs (miRNAs, miRs) are small non-coding RNAs that are photogenically conserved and act as master regulators of gene expression [84]. miRNAs were predicted using the web platform miRNet 2.0 (https://www.mirnet.ca/, version 2.0, accessed on 22 July 2022) [62]. For our analysis, we used the genes that were differentially expressed in the respective analyses. The predicted miRNAs for all three analyses (progeria DEGs, aging DEGs, and the 157 common DEGs) and the subsequent analyses are available in the Supplementary Materials (Table S4). Here, we focus on the miRNA prediction using the 157 common DEGs, resulting in 37 predicted miRNAs.

The calculated network of these miRNAs and their interaction partners were imported to Cytoscape for further analysis and filtered for DEGs. The three common aging-related DEGs (WNT16, IGFBP2, and UCP2) revealed five predicted miRNAs: WNT16 is associated with one miRNA (hsa-mir-181a-5p, human-microRNA-181a-5p), UCP2 is associated with two miRNAs (hsa-mir-26a-5p and hsa-mir-124-3p), and IGFBP2 is associated with three miRNAs (hsa-mir-124-3p, hsa-mir-126-3p, and hsa-mir-27b-3p). The same five miRNAs were predicted for aging and progeria (Supplementary Materials, Table S4 and Figures S6–S8).

**Figure 7:**
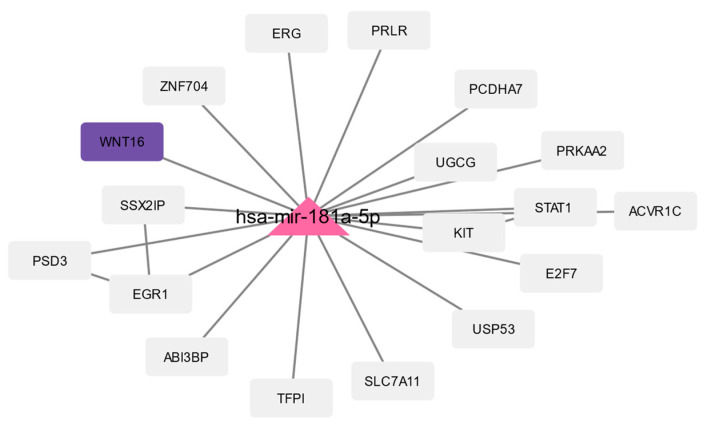
miRNA prediction and network visualization of miRNAs related to WNT16. Predicted miRNA interaction partners of hsa-mir-181a-5p, WNT16 is highlighted.

**Figure 8:**
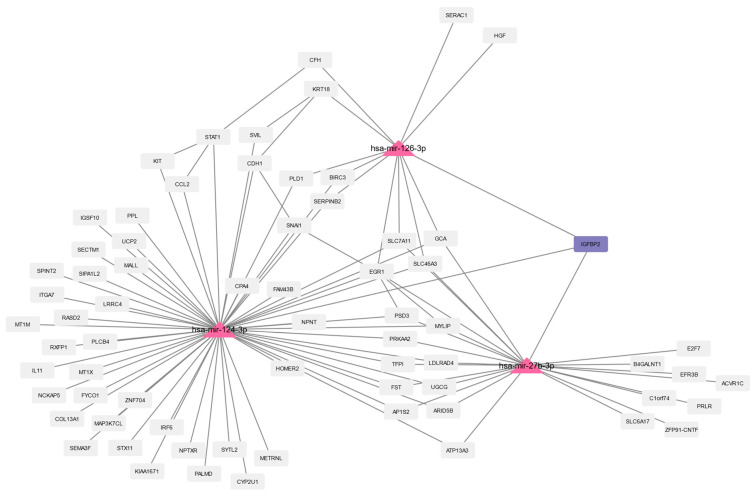
miRNA prediction and network visualization of miRNAs related to IGFBP2. Predicted miRNA interaction partners of hsa-mir-124-3p, hsa-mir-126-3p, and hsa-mir-27b-3p, IGFBP2 is highlighted.

**Figure 9:**
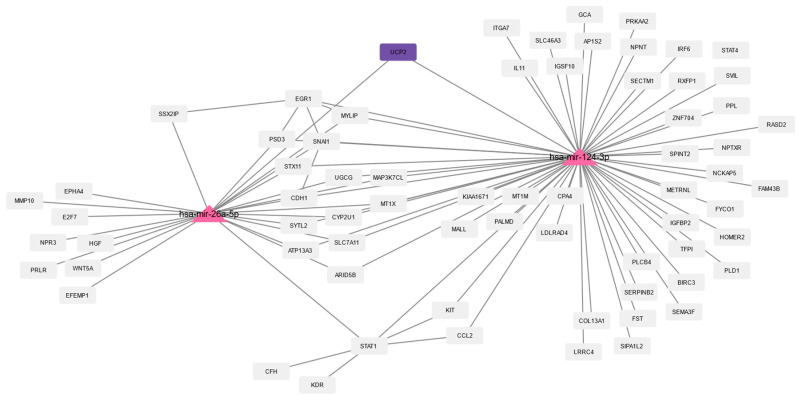
miRNA prediction and network visualization of miRNAs related to UCP2. Predicted miRNA interaction partners of hsa-mir-26a-5p and hsa-mir-124-3p, UCP2 is highlighted.

The miRNAs and their interaction partners of the 157 common DEGs are visualized in Figure 7, Figure 8 and Figure 9. Figure 7 shows hsa-mir-181a-5p and its interaction partners, with WNT16 being highlighted. The interaction partners of the three miRNAs associated with IGFBP2 (highlighted) are visualized in Figure 8, and Figure 9 shows hsa-mir-26a-5p and hsa-mir-124-3p, which are both predicted to interact with UCP2 (highlighted), and their interaction partners. In the Supplementary Materials (Figures S6–S8), we also visualize the interaction partners of these five miRNAs, including the changes in gene expression using the log2foldchanges obtained when comparing HGPS and healthy children and the interaction partners of the miRNAs and the changes in gene expression (log2foldchanges) obtained by comparing nonagenarians and healthy children.

### 3.5. Predicting Interactions Using NicheNet and Omnipath

Using NicheNet [64] and the Omnipath database [63,85], we combined the experimental results regarding RNA expression obtained from the dataset by Fleischer et al. [19] with prior knowledge regarding potential interaction partners from the Omnipath database.

**Figure 10:**
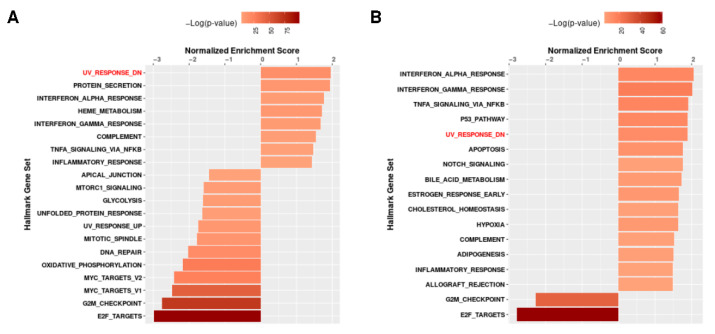
NicheNet analyses of progeria and aging. (**A**) GSEA pathways when comparing nonagenarians and healthy children. (**B**) GSEA pathways when comparing HGPS patients and healthy children.

Gene Set Enrichment Analysis (GSEA) [47] is integrated into the NicheNet workflow. Figure 10A,B visualizes the pathways in aging and HGPS, respectively. The pathway UV response (processes resulting in changes in a cell or organism upon ultraviolet radiation/UV light) has the highest positive normalized enrichment score (NES) in aging and is also among the top five positive enriched pathways in progeria. Here, we focus on UV response, as it has been shown that sun exposure induces the expression of progerin in human skin [86]. Additionally, accumulation of progerin has been associated with vascular disease in progeria [87], but, over time, it also accumulates in non-HGPS individuals [88]. It might thus contribute to vascular aging and vascular disease [88].

**Figure 11:**
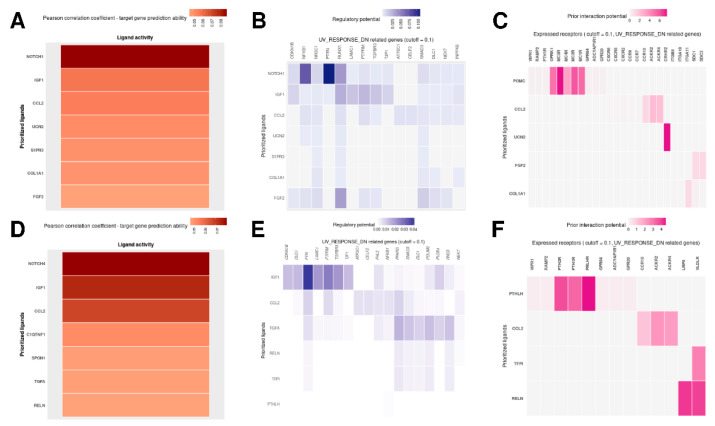
NicheNet analyses of progeria and aging. (**A**) Pearson correlation of the predicted ligands in the aging comparison. (**B**) Heatmap of predicted ligand–target interactions in the aging comparison. (**C**) Heatmap of the predicted ligand–receptor interactions in the aging comparison and their respective receptors. (**D**) Pearson correlation of the predicted ligands in the progeria comparison. (**E**) Heatmap of predicted ligand–target interactions in the progeria comparison. (**F**) Heatmap of the predicted ligand–receptor interactions in the progeria comparison.

The subsequent NicheNet analyses to predict the potential ligand–receptor pairs were performed with UV response as the pathway of interest (Figure 11B,C,E,F). The Pearson correlation of the predicted ligands involved in UV response is shown in Figure 11A,D. Darker color indicates a higher prediction ability. The target genes for these ligands regulating genes related to UV response are visualized as heatmaps for both groups in Figure 11B (comparison nonagenarians and healthy children) and Figure 11E (comparison progeria patients and healthy children). The color intensity indicates the regulatory potential for the top-ranked targets (the 0.1 quantiles) with targets according to the prior model, which was derived from prior knowledge archived in the Omnipath database.

**Figure 12:**
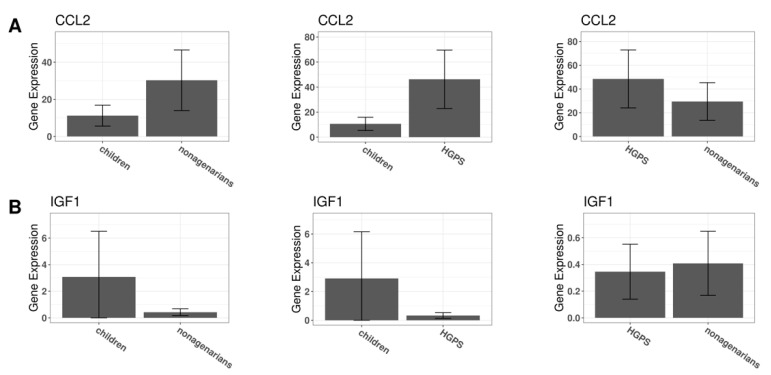
NicheNet analyses of progeria and aging. (**A**) Expression of CCL2 in children and nonagenarians, children and progeria patients, and progeria patients and nonagenarians. (**B**) Expression of IGF1 in children and nonagenarians, children and progeria patients, and progeria patients and nonagenarians.

Figure 11B,E shows the predicted ligand–target interactions. Both analyses have IGF1 (insulin-like growth factor 1) and CCL2 as common ligands for the predicted target genes. IGF1 expression is higher in children compared to nonagenarians and progeria patients. When comparing IGF1 expression in nonagenarians and progeria patients, the expression levels show little difference (Figure 12B).

Predicting the ligand–receptor interactions shows which of the receptors that are expressed in the respective genes might interact with the prioritized ligands. Figure 11C shows the comparison of nonagenarians and healthy children focusing on UV response, Figure 11F shows the comparison of progeria patients and healthy children focusing on UV response.

The only ligand that both analyses have in common is CCL2. CCL2 is an upregulated DEG when comparing nonagenarians and healthy children. It is upregulated even more when comparing progeria patients and healthy children. When comparing CCL2 expression in progeria patients and nonagenarians, CCL2 is more expressed in progeria (Figure 12A).

ACKR4 is the only potential CCL2 receptor that is also a DEG in aging and progeria.

**Figure 13:**
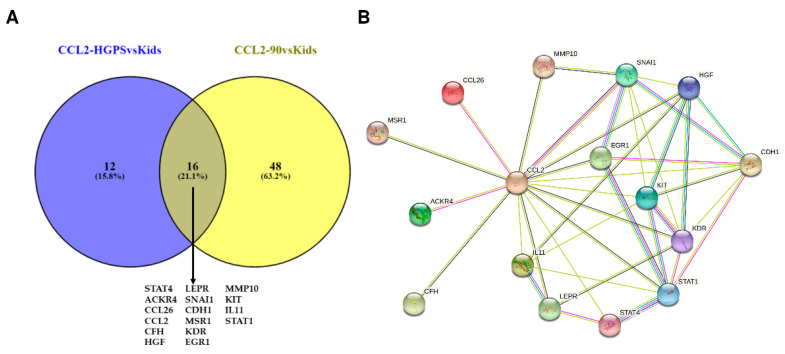
NicheNet and STRING analysis of progeria and aging. (**A**) The 16 common CCL2 interaction partners are the overlapping neighbors of CCL2 in the DEGs of the progeria comparison (blue) and the DEGs of the aging comparison (yellow), respectively. (**B**) STRING network of CCL2 and the 16 CCL2 interaction partners that are differentially expressed in both comparisons.

To find possible interaction partners of CCL2 that are differentially expressed in both analyses, we uploaded the set of HGPS DEGs obtained by comparing progeria patients and healthy children in STRING. The results of the STRING analysis, the interactions found between the DEGs, were subsequently analyzed in Cytoscape by selecting CCL2 and its neighbors, resulting in a list of DEGs (CCL2-HGPSvsKids, blue circle in Figure 13A). The same steps were repeated using the aging DEGs obtained by comparing nonagenarians with healthy children (CCL2-90svsKids, yellow circle in Figure 13A). The overlapping 16 DEGs of both groups (STAT4 (Signal Transducer And Activator Of Transcription 4), ACKR4, CCL26, CCL2, CFH (Complement Factor H), HGF, LEPR (Leptin Receptor), SNAI1 (Snail Family Transcriptional Repressor 1), CDH1 (Cadherin 1), MSR1 (Macrophage Scavenger Receptor 1), KDR, EGR1 (Early Growth Response 1), MMP10 (Matrix Metallopeptidase 10), KIT, IL11 (Interleukin 11), and STAT1) were visualized in STRING (Figure 13B).

The STRING analysis shows that all of the DEGs that are part of both analyses have already been associated with CCL2. Several of these DEGs have at least been co-mentioned in PubMed abstracts: Four (KIT, CDH1, STAT4, and IL11) have been co-mentioned in PubMed abstracts, four (MSR1, LEPR, EGR1, and STAT1) have been co-mentioned and have putative homologs that are co-expressed in other organisms, four (HGF, CFH, MMP10, and KDR) have been co-mentioned and are co-expressed in humans, and one (SNAI1) has been co-mentioned, is co-expressed in humans, and has been associated with CCL2 in experimental/biochemical data. CCL2 and both CCL26 and ACKR4 (atypical chemokine receptor 4) have been co-mentioned and have experimental/biochemical data suggesting a possible functional link.

Out of the three likely interaction partners of CCL2 (CCR10 (C-C chemokine receptor type 10), ACKR2 (atypical chemokine receptor 2), and ACKR4) that were predicted using NicheNet and focusing on UV response, only ACKR4 is differentially expressed in both analyses. As ACKR4 was regulated in different directions in progeria and aging (downregulated in HGPS, upregulated in aging, Figure 3C–E), ACKR4 might play an important role in both processes and might be involved in the severity of the symptoms or the differences between accelerated and normal aging.

### 3.6. Proteomics

Multi-omics integration analysis offers additional information and thus a better understanding of the potential progeria- and aging-related markers. Although RNA and protein are closely related, it has become apparent that protein levels are controlled by other factors besides mRNA levels [89]. For a complete understanding of the regulation of gene expression, integrating both transcriptomics and proteomics is necessary [89].

Therefore, we analyzed the literature on proteomics in aging research and compared the findings of proteomics analyses with our RNA-Seq analysis results.

Johnson and colleagues (2020) performed a systematic review of 36 different proteomics analyses regarding human aging involving more than 11,000 participants [90]. They report that 1128 proteins were reported as significantly changing with age by at least two different studies, and 66.58% of these proteins were reported in two or more different cell types and/or tissues [90]. Among these proteins was IGF1, found in cerebrospinal fluid and plasma and associated with longevity via the insulin-IGF1 signaling pathway [90].

Thirty-two proteins were even reported by at least five or more analyses and have known connections to age-related diseases and aging, including HGF, which has been shown to attenuate inflammation and severity of pulmonary artery hypertension in a rat model [90].

To crosscheck our RNA-Seq DEGs, we compared our DEGs to the proteins resulting from Johnson and colleagues’ meta-proteomics analyses. According to Johnson et al. (2020), four of our DEGs of special interest (CCL2, IGF1, IGFBP2, and KRT18) were reported as aging-related proteins [90].

Moaddel et al. (2021) have remarked that changes in plasma protein levels do not necessarily affect the protein level in another tissue or matrix [91]. Hence, they focused on proteins with plasma concentrations significantly associated with age (in at least two studies) that were additionally associated with age in at least one non-plasma matrix [91]. Applying these stricter standards, three of the genes that are differentially expressed in aging and progeria (TFPI, STAT1, IGFBP2) appear to be associated with changes in protein expression.

Tsitsipatis and colleagues (2022) analyzed the proteins of primary skin fibroblasts of healthy donors between 22 and 89 years of age and highlighted the pathways playing a key role in skin fibroblast aging [92]. As they created an ex vivo model by cultivating the fibroblasts, not all traits of skin aging were faithfully recapitulated [92]. However, several previous studies and their internal tests measuring collagen expression indicate the value of ex vivo fibroblast models [92]. They generated a comprehensive proteome of skin fibroblasts, which can be used to crosscheck whether DEGs revealed by RNA-Seq data analysis also indicate changes in the proteome.

**Figure 14:**
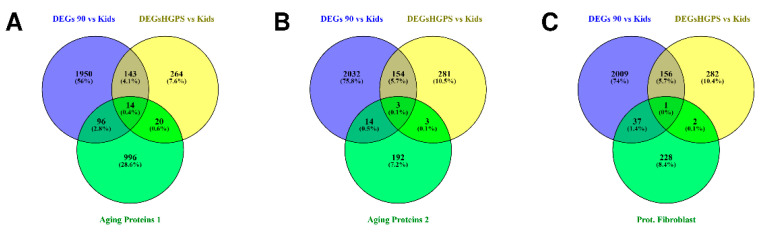
Venn diagram analyses of progeria and aging in our RNA-Seq results and proteomics literature. (**A**) The DEGs of the aging comparison (blue) and the DEGs of the progeria comparison (yellow) compared with the proteins of the proteomics analyses reviewed by Johnson et al. (2020; available in their Supplementary Material [90]; green). (**B**) The DEGs of the aging comparison (blue) and the DEGs of the progeria comparison (yellow) compared with the proteins of the proteomics analyses reviewed by Moaddel et al. (2021; available in their Supporting Information [91]; green). (**C**) The DEGs of the aging comparison (blue) and the DEGs of the progeria comparison (yellow) compared with the proteins of the proteomics analyses by Tsitsipatis et al. (2022; available in their Supporting Information [92]; green).

Figure 14 summarizes the comparison of our RNA-Seq data analyses and the proteomics analyses: According to the review by Johnson and colleagues (2020), the respective proteins of 110 DEGs of the aging comparison (Figure 14A, blue circle) and 34 DEGs of the progeria comparison (Figure 14A, yellow circle) were found during proteomics analyses (Figure 14A, green circle, data available in the supplementary materials of Johnson et al.’s (2020) publication [90]). Fourteen of these DEGs are common DEGs in aging and progeria (Figure 14A, Table 2 “Aging Proteomics 1”). When applying the stricter definition of Moaddel et al. (2021; Figure 14B, data available in the supplementary data of Moaddel et al.’s (2021) publication [91], green circle), there remained 17 aging-related DEGs (Figure 14B, blue circle) and 6 HGPS-related DEGs (Figure 14B, yellow circle). Three of these DEGs (Figure 14B, Table 2 “Aging Proteomics 2”) are associated with both aging and progeria.

Comparing the results of Tsitispatis analysis with our DEGs (Figure 14C) indicates that several of the observed changes appear to affect the protein expression: 38 of the aging-related DEGs (Figure 14C, blue circle) and three of the progeria-related DEGs (Figure 14C, yellow circle) overlap with aging-related proteins of Tsitsipatis and colleagues’ proteomics analysis (Figure 14C, data available in the supplementary materials of Tsitsipatis et al.’s (2022) publication [92], green circle). Of these DEGs, WNT5A was significantly differentially expressed in all three groups (Figure 14C, Table 2 “Proteomics Fibroblasts”).

### 3.7. Validation Using a Different RNA-Seq Dataset

Mateos and colleagues (2018) combined RNA-Seq and High-Resolution Quantitative Proteomics (iTRAQ, isobaric tag for relative and absolute quantification) using two biological replicates for analyzing fibroblast cell lines derived from progeria patients and healthy parental controls [107]. The iTRAQ technique uses mass tags to label peptides allowing the combination of time points or replicates, which improves the identification of low levels of a protein [108]. The technique can also be used to analyze phosphorylated proteins [108]. In their study, Mateos et al. (2018) performed RNA-Seq and iTRAQ using their own fibroblasts, which were derived from donors suffering from progeria and their parents [107]. The group aimed to find molecular pathways affecting premature aging [107]. After analyzing the significant transcripts and proteins, they focused on ribose-phosphate pyrophosphokinase 1 (PRPS1), which affects the purine metabolism and is significantly decreased in HGPS compared to healthy parental controls [107].

**Figure 15:**
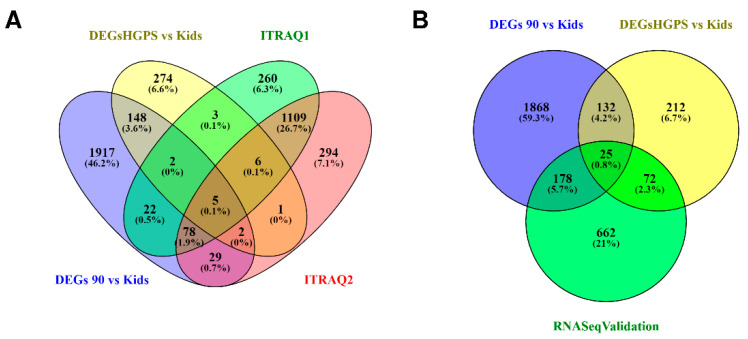
Venn diagram analysis of progeria and aging regarding transcriptomics and proteomics. (**A**) The DEGs of the aging comparison (blue circle) compared with the DEGs of the progeria comparison (yellow circle) and compared with the proteins of the two biological replicates (ITRAQ1 (green) and ITRAQ2 (red circle)) analyzed by Mateos and colleagues (2018, available in their Supporting Information [107]). (**B**) Comparison of the DEGs of the aging comparison (blue) and the DEGs of the progeria comparison (yellow) compared with the DEGs of the RNA-Seq analysis by Mateos et al. (2018; available in their Supporting Information [107]; green circle).

To validate our results, we compared Mateos et al.’s (2018) RNA-Seq analysis results with our analyses. Our comparison of progeria patients and healthy children and their comparison of progeria patients and their parents have 97 genes in common (Figure 15B, overlap of the yellow and the green circle) and our comparison of nonagenarians and healthy children results in 203 common genes with Mateos and colleagues’ results (2018; Figure 15B, overlap of the blue and the green circle). Among these common genes are KRT18, WNT5A, IGFBP2, EGR1, and ADAMTS15.

Comparing our RNA-Seq DEGs with the results of Mateos and colleagues’ proteomics analysis (Figure 15A) also reveals some DEGs that appear to affect protein levels, including PLCB4, BST1, STAT1, IGFBP2, and SERPINB2 (overlap of all four ellipses in Figure 15A, Table 3), even though the respective analyses were done with different datasets.

## 4. Discussion

In 1987, Rowe and Kahn proposed the concept of “successful aging”, pointing out that many of the changes regarded as “normal” during aging are preventable [109]. They also reported that some of these changes could be reversed [109] by interpreting “aging as a disease”, a notion that has already been proposed in ancient times [110] and has recently garnered attention [110,111]. Regardless of whether aging should be seen as a disease or not, extending not only the lifespan but also the health span [1,110,111] and possibly even rejuvenation [28] are of great interest. In this study, we analyzed a publicly available RNA-Seq dataset and proteomics data using bioinformatic tools.

Although the name “progeria” is derived from Greek for “prematurely old” [7], there are differences in differential gene expression between HGPS and “normal” aging. Gene expression in both groups, the HGPS patients and the nonagenarians, differs from gene expression in the control group of healthy children. However, there are distinct differences between the analysis results for progeria and aging. While both conditions have 157 DEGs in common, there are also DEGs specific to the respective conditions. This results in different biological processes being affected by the changes in gene expression, evident in enrichment analyses.

The differences might be due to differences between progeria patients and nonagenarians. While progeria patients are children suffering from a rare and fatal disease resulting in premature aging [112], the nonagenarians might be examples of “successful aging” [109].

Additionally, enrichment analyses might reveal relevant information for understanding and treating the conditions.

For instance, the only hallmark gene set that is enriched when comparing progeria patients and healthy children is KRAS signaling up. KRAS is known as the most frequently mutated RAS isoform [113]. Due to the oncogenic nature of mutations in the RAS genes, RAS inhibitors such as farnesyltransferase inhibitors (FTIs) have been researched as potential anticancer drugs [113,114], although FTIs did not advance into clinical use due to their lack of efficacy in cancer therapy in clinical trial [114,115]. The similarity in the post-translational processing of RAS and progerin led to the repurposing of FTIs as potential treatments for HGPS [115]. One of these drugs, lonafarnib (zokinvy), was successfully tested as progeria treatment in the first clinical trial for treating progeria [15] and has since been FDA approved [17], becoming the first FDA approved drug for progeria treatment [115].

Since Fleischer et al. (2018) obtained the dermal fibroblasts from “apparently healthy individuals” via the Coriell Institute cell repository [19], there is no further information available about the donors. The number of samples (5–7/group) is sufficient for RNA-Seq data analysis using DESeq2 [41,116]. However, a bigger sample size and detailed medical history of the fibroblast donors could have contributed to further insights.

Novelty of our findings in a nutshell: Here, we focused on the 157 common DEGs in progeria vs. aging speculating they should be involved in or at least related to the aging process and might be the key to understanding aging and the processes involved. This comparative aspect of our study is completely new though the dataset by Fleischer et al. [19] has been analyzed in other directions before (see Table 1). By this comparison, we can much better isolate the physiological genes for high age and separate them from the accelerated, pathological aging in progeria. More importantly, all these genes are markers for specific pathways: downregulated in high age are KRT8, KRT18, ADAMTS15, ACTN4P1, and UCP2 whereas ACKR4, WNT16 and IGFBP2 are upregulated in nonagenarians. Hence, this study is novel to reveal by an extensive comparative analysis over multi-omics datasets pathways involved in achieving a very high age separated from pathological aging pathways. Definitely this is only a first start for more extensive analyses including extensive cell biology experiments on the different pathways involved (see also below: limitations). In particular further analysis has to find out, how far the changes in pathways and gene expression found in nonagenarians are markers or makers of successful aging. This will be critical for any therapeutic strategies to be derived from the analysis. We are next discussing the individual findings:

Surprisingly, six of these DEGs are regulated in different directions in progeria and aging, respectively. Five of the DEGs (KRT8, KRT18, ADAMTS15, ACTN4P1, and UCP2) are upregulated in HGPS patients compared to healthy children and nonagenarians but downregulated when comparing nonagenarians and healthy children.

The sixth DEG, ACKR4, is downregulated in children suffering from progeria compared to healthy children and nonagenarians. Nonagenarians have higher ACKR4 levels than healthy children. The opposite regulation of these genes might lead to a better understanding of the differences between progeria and aging. Furthermore, the regulation of the DEGs could indicate accelerated aging or normal aging.

KRT8 (Keratin 8) and KRT18 (Keratin 18) have been linked to modulating cellular stress response and cell resistance to apoptosis [117]. KRT18 has been suggested as a possible biomarker for frailty and aging [118], due to KRT18 and cKRT18 (caspase-cleaved fragment of keratin 18 (KRT18)) being biomarkers for diseases with apoptotic and mitochondrial defects, which are among the hallmarks of aging, and its association with senescence and anti-mitochondrial auto-antibody formation [118].

The nonagenarians, on the other hand, appeared to have rather low KRT18 expression, although KRT18 levels would be expected to rise with increasing age [118]. If KRT18 expression in fibroblasts is similar to other tissues, this finding might indicate that the nonagenarian fibroblast donors were of extremely good health or that progeria severely affects the skin, which corresponds to skin problems being among the typical progeria symptoms [10,11]. Interestingly, Gill and colleagues (2022) reported that KRT8 and KRT18 were among the genes they observed as downregulated in aging and upregulated upon transient reprogramming, their method for cell rejuvenation [28]. Therefore, examining KRT18 and its interaction partners in HGPS and different age groups might lead to further insights regarding aging and “successful aging”.

ADAM (a disintegrin and metalloproteinase with thrombospondin motifs) metallopeptidase with thrombospondin type 1 motif 15 (ADAMTS15) is upregulated in HGPS but appears to be downregulated upon aging (both compared to healthy children). ADAMTS15, along with ADAMTS1, 4, 5, 9, and 20, is involved in several processes, including palate formation, skin pigmentation, myogenesis, and cardiac development [119], all of which appear to be affected by progeria. In addition, ADAMTS15 is involved in the turnover of cartilage and/or bone during joint inflammation [120] and the inverse correlation of ADAMTS15 and CITED2 (Cbp/P300 Interacting Transactivator With Glu/Asp Rich Carboxy-Terminal Domain 2) expression links it to the Wnt pathways associated with bone formation and inflammatory arthritis [120].

Thus, ADAMTS15 might be an interesting research target in progeria and aging-related research, especially as WNT16 is among the only three DEGs that progeria, aging, and the aging pathway have in common.

The role of actinin alpha 4 pseudogene 1 (ACTN4P1) is not yet known. Pseudogenes have long been regarded as “void of function” [121] or “junk DNA” [122,123]. However, research has shown that they can affect coding genes and are transcribed into RNA [122,123] and are involved in regulatory functions [123].

Progeria patients had higher ACTN4P1 expression than healthy children in our study and even higher ACTN4P1 expression compared to nonagenarians. To our knowledge, this is the first publication mentioning ACTN4P1, which warrants further investigation of which processes and interaction partners are affected by ACTN4P1 and whether the pseudogene is involved in aging-related processes.

WNT16 is among the DEGs with rather drastic changes in gene expression, indicating that WNT16 might be of special importance in both progeria and aging. This is coherent with literature as Marthandan and colleagues (2016) assessed the five most commonly used human fibroblast strains for laboratory use by deep RNA sequencing and real-time PCR and demonstrated that WNT16 and IGFBP2 are among the most differentially expressed genes upon aging [124]. In aging research, WNT16 has already garnered interest due to its association with bone mineral density, bone strength, and fracture risk [125]. WNT16B has been associated with regulating the onset of replicative senescence and belongs to the WNT family, a family of secreted proteins involved in the development, aging, senescence, and tumorigenesis [126]. Additionally, it has been proposed that progerin directly affects the transmission of Wnt (Wingless/Integrated) signaling pathway, which is known to be impaired in HGPS [127]. Our study further confirms a possible connection between Wnt signaling, progeria, and aging.

Another aging-related gene, UCP2 (Uncoupling Protein 2), is shown as upregulated in progeria compared to healthy children and nonagenarians. Upregulation of UCP2 was observed in aged rats [128] and a mouse model of premature aging [129,130], where UCP2 expression appeared to have metabolic effects [130]. Its upregulation in spontaneously obese mice suggested UCP2-mediated metabolic adaption to the increase of fatty acid biosynthesis and elevated lipid levels [128]. Increased UCP2 expression has been correlated with increased levels of free fatty acids, which are proposed to be involved in downregulating IGF1 levels via a negative feedback loop [128]. Therefore, UCP2 has been associated with counterregulatory effects on aging and age-related pathologies in mice, possibly via modulating the insulin/IGF1 signaling pathway, which indicates that a targeted increase of UCP2 levels might prolong the lifespan of mammals [128].

However, in progeria, the high UCP2 levels do not correlate with patient’s body weight, as low body weight is one of the characteristics of progeria [11]. In addition, there appear to be parallels between UCP2 and the different LMNA isoforms. A study comparing the effects of lamin A, lamin C, and progerin, the truncated form of lamin A, in mice revealed that progerin and lamin C regulate mitochondrial biogenesis and energy expenditure via triggering antagonistic signals in adipose tissue [131]. While mice only expressing lamin C were obese and had an increased lifespan, the role of progerin in adipose tissue homeostasis might have an opposing effect on lifespan [131]. Additionally, the rather skinny progerin-expressing mice were more sensitive to insulin and appeared to have a higher metabolic rate and use more carbohydrates [131]. In contrast, the lamin C-expressing mice were moderately insulin-resistant, showed reduced overall energy consumption, and appeared to prefer fatty acids [131]. The involvement of UCP2 in aging and insulin signaling is similar to progerin and is of great interest to research further.

Insulin-like growth factor-binding protein 2 (IGFBP2) levels positively correlate with age, and insulin sensitivity and inversely correlate with the body mass index (BMI) [132]. Van den Beld et al. (2018) conducted a 20-year longitudinal study, repeatedly measuring BMI, IGF1, IGFBP2, insulin sensitivity, and mortality around the ages of 55, 65, and 75 in 539 participants [132]. They reported, when adjusted for BMI, IGFBP2 levels and insulin sensitivity show a positive correlation [132]. Therefore, the authors suggest IGFBP2 as a possible marker for insulin sensitivity [132].

We are, to our knowledge, the first to report and stress these elevated IGFBP2 levels in progeria patients. In our comparative analysis, IGFBP2 levels in healthy children and nonagenarians show its upregulation with age. However, in progeria, IGFBP2 expression is considerably more elevated than in nonagenarians suggesting its role in both aging and progeria. Such upregulation in progeria could be related to their weight, as children suffering from progeria typically have rather low BMIs [11]. The age-related increase of IGFBP2 levels, especially after 50 [132], could also be connected with the high IGFBP2 expression in accelerated aging and in serum, it appears to be a mortality marker that positively correlated with insulin sensitivity [132].

While pseudogenes such as ACTN4P1 are still garnering research interest [121,122,133,134], microRNAs (miRNAs/miRs), which have been equally disregarded for a long time [135], are increasingly recognized as therapeutic targets [135] and show promising therapeutic results [136]. Hence, we included miRNA prediction, which is a promising research field on its own, in our analyses.

Three of the 37 miRNAs that were predicted using the common DEGs as input might be associated with IGFBP2: hsa-mir-27b-3p, hsa-mir-126-3p, and hsa-mir-124-3p.

In the plasma, hsa-mir-126-3p appears to be upregulated with age [137], while the miRNA was found to be downregulated in blood samples of centenarians and has therefore been proposed as a potential longevity biomarker [137,138]. Olivieri et al. (2014) reported that the increase of hsa-mir-126-3p blood level was accompanied by an increase of hsa-mir-126-3p in human endothelial cells during senescence [139]. They also observed lower hsa-mir-126-3p levels in type 2 diabetes mellitus patients and proposed a possible interrelationship between miR-126-3p (microRNA-126-3p) downregulation and age-related conditions with a pro-inflammatory background, while an increase of mir-126-3p might act as a positive compensatory mechanism [139]. miR-27b expression appears to affect wound healing in the skin, as a study by Bi et al. (2020) indicates [140]. They reported increased fibroblast proliferation and thus accelerated healing of scald wounds in rats upon miR-27b inhibition [140].

Both IGFBP2 and UCP2 are associated with miR-124, which has been shown to increase in senescent skin and upon UVB-irradiation (type B ultraviolet), indicating a possible role of miR-124 in UVB-induced skin aging [141]. UCP2 is also associated with miR-26a-5p. Measured in serum, miR-26a could serve as a prognostic marker for osteoporosis and appears to regulate serum IGF1 levels in osteoporosis patients [142]. Additionally, miR-26a-5p has been linked with UVB-induced apoptosis [137]. Increased expression of miR-181a, which was predicted to be associated with WNT16, has been reported upregulated in keratinocytes undergoing replicative senescence [137]. Furthermore, miR-181a is among the biomarkers of aging expressed by dermal fibroblasts and has been linked with skin immunosenescence and the age-related inflammatory phenotype in CD4+ T cells (CD4-positive cells/T helper cells) [137].

While GO enrichment focuses on DEGs, a GSEA analysis takes the whole gene set into account. Thus, using NicheNet analysis, GSEA, the Omnipath database, and the well-known GSE113957 dataset, we present here to our knowledge the first integrated data analysis of the pathways involved in aging and progeria. Furthermore, we show genes and their interaction partners involved in these pathways.

According to our analysis, UV response has the highest positive normalized enrichment score when comparing nonagenarians and healthy children. Additionally, UV response is among the top five pathways when analyzing progeria. Lesiak et al. (2017) assessed the progerin expression upon sun exposure in vivo and demonstrated that one week of sun exposure was enough to significantly elevate the progerin levels in the skin of participants in their twenties almost to the amount of progerin measured in elderly participants (64.1 ± 13.1 years) with photoaged skin [86]. Due to this experimentally backed correlation between UV exposure and progerin expression, we decided to focus on UV response in our NicheNet analyses. In both analyses, aging and progeria, IGF1 and CCL2 are among the prioritized ligands.

IGF1 (insulin-like growth factor 1) has been associated with IGFBP2 [132,143] and controls apoptosis [143,144]. Van den Beld and colleagues (2018) studied IGFBP2 and IGF1 concentrations, as well as insulin sensitivity and BMI in a 20-year longitudinal study and concluded that IGFBP2 levels can predict mortality if interpreted in relation to insulin sensitivity [132]. Hu et al. (2009) also reported a correlation between high IGFBP2 levels and mortality in subjects older than 70 years [143]. They proposed a possible association between low IGF1 expression, which is associated with lower mortality in animal models, and low IGFBP1 (Insulin Like Growth Factor Binding Protein 1) and IGFBP2 levels as markers for low IGF1 levels [143].

Fibroblasts of nonagenarians express less IGF1 than fibroblasts of healthy children, and IGF1 is indeed a DEG in natural aging. In fibroblasts donated by progeria patients, IGF1 appears to be slightly lower expressed than in nonagenarians. When comparing progeria patients and healthy children, IGF1 is also lower expressed.

As age appears to have a greater effect on UV-induced damages than skin type [145], the effects of increased IGF1 expression were studied [146,147]. In aged skin, exogenous IGF1 [147] as well as dermabrasion and sun-protected skin-healing, which increased IGF1 levels [146], were found to restore the response to UVB radiation [146,147]. Therefore, it would be of interest whether treatments affecting IGF1 expression influence progeria-related skin abnormalities.

Additionally, UV response might link inflammation and aging, “inflammaging”, as exposure to UV light is a well-known method to provoke inflammation [148], and CCL2 expression is also induced by inflammatory stimuli [149]. The C-C Motif Chemokine Ligand 2 (CCL2), which is also known under several other names, including monocyte chemoattractant protein-1 (MCP-1), is the other prioritized ligand aging and HGPS have in common when analyzed regarding UV response. Among its predicted interaction partners are ACKR4, the atypical chemokine receptor 4 that is also known as CCR11, and a variety of other names. ACKR4 is also among the CCL2-related DEGs that progeria and aging have in common. Although CCL2 levels are higher in progeria patients than in nonagenarians, both nonagenarians and progeria patients have higher CCL2 levels than healthy children.

Again, the individual facets are known, but the suggested synthesis sheds new light on this aging pathway. Since increased CCL2 levels have been associated with inflammation and aging, Luciano-Mateo and colleagues (2020) crossbred mice bearing a mutation in their LMNA gene with mice overexpressing CCL2 [150]. The combination of accelerated aging and CCL2 overexpression significantly reduced the lifespan and the health span of the mice [150]. Additionally, higher CCL2 levels appeared to worsen accelerated aging and also affected the energy metabolism and the 1-C metabolism, as well as the mitochondrial function of the mice bearing both the LMNA mutation and CCL2 overexpression [150].

These results, as well as our observations, suggest CCL2 as an additional target in aging and progeria research. Therefore, we visualized the interactions of CCL2 and the CCL2-related DEGs involved in both aging and progeria. In this study, we focus on the interaction between CCL2 and ACKR4, as both are involved in the UV response pathway, which we selected as an example for NicheNet analysis. ACKR4 has been mentioned as a receptor for CCL2 [151] which is upregulated in nonagenarians compared to healthy children in our study, whereas the ACKR4 expression in progeria is very low in all comparisons. MCP-1/CCL2 can bind to a common binding site on ACKR4/CCR11 [151]. However, the relationship between CCL2 and ACKR4 is not yet explored. Hence, we predict that exploring the interactions of ACKR4 and CCL2 in aging in future research will be rather interesting.

Although we elaborated only on UV response as an example, the other pathways suggested by NicheNet analysis are equally interesting, and focusing on the ligands and receptors involved might generate further insights regarding aging and progeria. Due to the plethora of information contained in RNA-Seq experiments, reanalyzing existing RNA-Seq data can still generate new insights. Even if in silico analysis can offer great insights and help generate new hypotheses, subsequent in vitro and in vivo studies are necessary to further validate the targets found using omics analyses.

Further validation for RNA-Seq analysis results is analyzing the protein expression measured via proteomics analyses. As mRNA levels and protein levels only modestly correlate, gene expression is also controlled by post-transcriptional regulation [89]. Thus, changes in gene expression observed in RNA-Seq data do not necessarily result in changes in protein expression [89]. Crosschecking transcriptomics and proteomics results can therefore validate whether the DEGs found via RNA-Seq data analysis are likely to affect the organism. For analyzing laboratory results, a multi-omics approach, such as the combination of transcriptomics and proteomics analyses performed by Mateos et al. (2018) [107], can generate further insights.

After additionally analyzing aging- and progeria-related proteomics analyses, the importance of STAT1, which is differentially expressed in both comparisons (nonagenarians and healthy children and HGPS patients and healthy children) and in proteomics analysis, became apparent. Signal Transducer And Activator Of Transcription 1 (STAT1) is involved in various pathways, including cell proliferation, differentiation, and apoptosis, and has been associated with cellular senescence [152]. In progeria, STAT1 has been shown to be involved in an interferon (IFN)-like response upon progerin-induced replication stress [153]. In turn, inhibition of STAT1 by calcitriol was demonstrated to improve the phenotypes of HGPS cells [153].

Among the genes that are differentially expressed in both of our RNA-Seq data analyses (aging-related and progeria-related), five genes were differentially expressed in the ITRAQ analyses performed by Mateos and colleagues (2018). Thus, the multi-omics approach shows that the changes in gene expression of these five DEGs (PLCB4, BST1, STAT1, IGFBP2, and SERPINB2) also affect protein expression (Table 3).

The meta-proteomics analysis of Johnson and colleagues (2020) further indicated that several of our DEGs appear to affect protein expression, as they were mentioned differentially expressed in at least two different proteomics analyses [90]. Besides our DEGs of special interest (IGFBP2 (mentioned three times), KRT18 (mentioned three times), CCL2 (mentioned twice), and IGF1 (mentioned twice)), several other DEGs (STAT1 (mentioned three times), TFPI (mentioned twice), HGF (mentioned five times), MSR1 (mentioned twice), EFEMP1 (mentioned four times), GDF5 (mentioned twice), KDR (mentioned twice), FST (mentioned twice), SECTM1 (mentioned twice), HS3ST3A1 (mentioned twice), and SPINT2 (mentioned three times)) have been found differentially expressed in proteomics analyses regarding aging. Additionally, a recent study by Tsitsipatis et al. (2022) indicates that Wnt5A expression [92] might be affected by changes in gene expression observed in our RNA-Seq data analyses.

Ikegami and colleagues (2020), who also analyzed Fleischer et al.’s (2018) dataset and samples from fibroblast cell lines, provided information on the pLMNA binding sites of various proteins in their supplementary data [22], and several of our DEGs of special interest (IGFBP2, IGF1, KRT8, KRT18, and CCL2) have been associated with the presence of gained pLMNA-binding sites.

Using Johnson et al.’s (2020) meta-analysis, we demonstrated that IGFBP2, IGF1, CCL2, and KRT18, which are differentially expressed in aging and progeria, have been observed as differentially regulated proteins in several aging-related proteomics analyses.

The tendencies in gene regulation of IGFBP2, IGF1, WNT16, UCP2, CCL2, KRT8, KRT18, and ADAMTS15 we observed in our analysis are confirmed by Mateos et al.’s RNA-Seq analysis (2018) of samples derived from progeria patients and their parents (Table 3). ACKR4 and ACTN4P1 were not among the genes in Mateos et al.’s RNA-Seq analysis (2018).

Analyzing the iTRAQ analysis results provided as supplementary data provided by Mateos and colleagues (2018) reveals that IGFBP2 is among the differentially expressed proteins when comparing progeria patients and their parents. This further highlights its potential importance in aging and progeria.

Such findings highlight the predictive value of multi-omics approaches such as the combination of RNA-Seq analysis and proteomics.

Limitations: Bioinformatics can indicate possible genes of interest and even model the effects of a pharmacological intervention [154]. However, one of the limitations of bioinformatics and modeling is that these theoretical predictions might differ from clinical results. One example of the limitations of theoretical predictions in clinical research is the use of FTIs as anti-cancer drugs, as described by Berndt et al. (2011) [113] and Xie and colleagues (2017) [114]. Despite promising predictions, the drugs did not have the desired clinical effect. Therefore, the use of bioinformatics needs to be combined with in vitro and in vivo experiments.

Nevertheless, bioinformatics and theoretical predictions can help save costs, resources, and time by predicting promising target genes and potential biomarkers. The approach of narrowing down potential genes of interest using publicly available datasets and subsequently confirming the hypotheses in the laboratory has led to discoveries such as the role of CD44 in diabetes [155,156]. It has been known for several years that the constantly growing number of publicly available datasets offers researchers the opportunity to repurpose these data and use them as the first step for their own research projects [155]. We would like to contribute to the broader use of this research approach by introducing and demonstrating the power of freely available bioinformatic tools.

The genes of interest we found using RNA-Seq analysis were confirmed by proteomics analyses, another “-omics” discipline, but before these results can be implemented in clinical practice, they need to be confirmed in the laboratory. Nevertheless, the likelihood of such validation is high in this case as indicated by additional bioinformatics validation of our results with multiple datasets.

Analyzing additional tissue types and blood samples might yield further insights as this study focused only on fibroblasts derived from donors in different age groups. All information and potential markers derived in this study are based on fibroblasts, another limitation of our study, as different cells and tissues might behave differently.

Increasing the number of samples for the different age groups would enhance the accuracy and reliability of the results, as increasing the sample sizes increases the reliability and reproducibility of gene set analyses [116,157].

In summary, we briefly introduced several omics methods for RNA sequence analysis that can be used on their own or in combination with both new data and already existing publicly available data. Here, we introduced some of the differentially expressed genes, their interaction partners, and their age-related implications, hoping to demonstrate some of the possibilities omics analyses may offer for aging research.

Finally, we would like to highlight the versatility of publicly available datasets such as the RNA-Seq dataset by Fleischer et al. that we used for our bioinformatics analyses. Since the raw data is available, every researcher can reanalyze the data using different tools and ask different questions. While using different tools might yield further insights, the real use of such datasets is repurposing the data for other research questions.

Although the dataset was already repurposed in several studies listed in PubMed, all of these studies produced different results. Our study still reveals new insights, although we intentionally used well-known high-quality bioinformatic tools.

As a novel approach for using the comprehensive dataset, we performed a comparative analysis of two RNA-Seq data analyses using subsets of the dataset (healthy children, nonagenarians, and progeria patients). By analyzing the RNA-Seq data of healthy children and nonagenarians, we found various DEGs related to normal aging. We also compared the RNA-Seq data of healthy children and children suffering from progeria, which resulted in various progeria-related DEGs. Further analysis revealed 157 common DEGs in both conditions, aging and progeria. This indicates the differences and similarities between aging and progeria. Subsequently, we focused on DEGs aging and progeria have in common and further validated our genes of interest using metaprotomics analyses. As our DEGs of interest have been found in aging-related proteomics analyses, the differences in gene regulation observed in the RNA-Seq analysis appear to affect the proteome as well, indicating the effect of the DEGs.

Interestingly, not all of the DEGs that both conditions have in common are regulated similarly. Some genes that are downregulated during aging are upregulated in progeria patients (KRT8, KRT18, UCP2, ADAMTS15, ACTN4P1) while others (ACKR4) are upregulated in nonagenarians but downregulated in children suffering from HGPS.

Despite progeria being known as premature aging, only three genes of the aging pathway are differentially expressed in nonagenarians and progeria patients compared to the same group of healthy children: WNT16, UCP2, and IGFBP2. We are—to our knowledge—the first to mention IGFBP2, which is known as an age-related mortality marker, as a potential biomarker in progeria. We also present here the miRNAs and interactomes for the three genes connecting aging, the aging pathway, and progeria: WNT16 (hsa-mir-181a-5p), UCP2 (hsa-mir-26a-5p and hsa-mir-124-3p), and IGFBP2 (hsa-mir-124-3p, hsa-mir-126-3p, and hsa-mir-27b-3p).

## Figures and Tables

**Table 1:** Comparison of the previous studies mentioning the use of the GSE113957 dataset [19] with the current analysis.

Study Title	Focus of the Study	Ref.
Progeria and Aging—Omics Based Comparative Analysis	Comparative analysis comparing two different RNA-Seq analyses (progeria vs. healthy children and nonagenarians vs. healthy children Focus on differentially regulated genes and Pathways in both normal aging and accelerated aging (progeria) First-time mention of the known age-related mortality marker IGFBP2 as a potential biomarker in progeria Presenting predicted miRNAs and interactomes for WNT16, UCP2, and IGFBP2 Metaproteomics analysis for a better understanding of the differentially expressed genes (DEGs) Multi-omics approach combining RNA-Seq data and proteomics data	[our study]
Epigenetic deregulation of lamina-associated domains in Hutchinson-Gilford progeria syndrome	Focused on epigenetic deregulation of lamina-associated domains (LADs) RNA-Seq data analysis was performed to investigate the contribution of the identified LAD-specific chromatin accessibility and DNA methylation changes on changes in gene expression Showed that ectopic progrin expression caused similar changes in gene expression as HGPS (examples given in their supplementary data: EDIL3 (EGF Like Repeats And Discoidin Domains 3), IGFBP7 (Insulin Like Growth Factor Binding Protein 7), POSTN (Periostin), NTN4 (Netrin 4), and IL13RA2(Interleukin 13 Receptor Subunit Alpha 2))	[21]
Phosphorylated Lamin A/C in the Nuclear Interior Binds Active Enhancers Associated with Abnormal Transcription in Progeria	Focused on Ser22-phosphorylated (pS22) Lamin A/C (pLamin) and its effect on gene regulation (mechanism) Used a different subset of Fleischer et al.’s dataset [19] (children and progeria patients aged between 2 and 17 years) Offer supplementary data indicating which of the genes dysregulated in progeria are affected by pLamin	[22]
Prevalent intron retention fine-tunes gene expression and contributes to cellular senescence	Focus on the alternative splicing type intron retention (IR) Investigated the role of global IR in cellular senescence Demonstrated that the splicing factor U2AF1 (U2 Small Nuclear RNA Auxiliary Factor 1) mediated IR of specific genes (e.g., CPNE1 (Copine 1)) contributed to cellular senescence The authors show that knockdown of U2AF1 slowed cell growth rate, increased CDKN2B (Cyclin Dependent Kinase Inhibitor 2B) expression, and decreased MKI67 (Marker Of Proliferation Ki-67), CDK1 (Cyclin Dependent Kinase 1), and CDK4 (Cyclin Dependent Kinase 4) expression	[23]
Analysis of transcriptional modules during human fibroblast ageing	Used Weighted Gene Co-expression Network Analysis (WGCNA) and found four natural aging- and one premature aging disease-associated modules Found aging-related pathways, including mitotic cell cycle, DNA replication, and DNA repair, as well as pathways related to essential cellular machineries (components of mitochondria, ribosome or RNA polymerase) to decline with age Found association of muscle and cardiovascular function-related pathways to HGPS Focused on LMNB1 (Lamin B1), KIFC1 (Kinesin Family Member C1), DLGAP5 (DLG Associated Protein 5), ANLN (Anillin, Actin Binding Protein), TACC3 (Transforming Acidic Coiled-Coil Containing Protein 3), LMNB2 (Lamin B2), DTYMK (Deoxythymidylate Kinase), ECM2 (Extracellular Matrix Protein 2), SVEP1 (Sushi, Von Willebrand Factor Type A, EGF And Pentraxin Domain Containing 1), PLSCR4 (Phospholipid Scramblase 4), KLHL24 (Kelch Like Family Member 24), SEMA3D (Semaphorin 3D), and TOR1AIP1 (Torsin 1A Interacting Protein 1) Reported CDK1, POLR2F (RNA Polymerase II, I And III Subunit F), SNAP23 (Synaptosome Associated Protein 23), UBE2D1 (Ubiquitin Conjugating Enzyme E2 D1), and MYL9 (Myosin Light Chain 9) as WGCNA hub genes KIFC1, DLGAP5, ANLN, ECM2, SVEP1, and TOR1AIP1 as potential aging biomarkers, and MYL9 to be related to progeria	[24]
Repetitive elements as a tran-scriptomic marker of aging: Ev-idence in multiple datasets and models	Focus on noncoding repetitive element (RE) transcripts as a transcriptomic marker of aging Age prediction using the 1,200 most significantly differentially expressed genes upon aging The authors report age-related RE transcript increases that might indicate biological age	[25]
Altered Chromatin States Drive Cryptic Transcription in Aging Mammalian Stem Cells	Focus on cryptic transcription and age-related changes in chromatin signatures During their study, McCauley et al. (2021) generated their own dataset (GSE156409) Fleischer et al.’s dataset [19] was among several GEO datasets the authors mentioned as one of the additionally analyzed datasets of the GEO database The authors looked at pathways regarding cryptic transcription changes and DNA methylation	[26]
Extremes of age are associated with differences in the expression of selected pattern recognition receptor genes and ACE2, the receptor for SARS-CoV-2: implications for the epidemiology of COVID-19 disease	Focus on genes encoding proteins known to interact with SARS-CoV-2 proteins (pattern recognition receptor genes and ACE2 (Angiotensin-converting enzyme 2)) Aimed to find a strategy for stratifying the risk of a severe COVID-19 infection Compared the oldest and the youngest age group but did not analyze progeria-related data Enriched KEGG (Kyoto Encyclopedia of Genes and Genomes) pathway analysis showed DEGs were involved in pathways including Cell Cycle and DNA replication Focused on age-related changes in PRR gene expression (e.g., TLR3 (Toll Like Receptor 3), TLR4 (Toll Like Receptor 4), NOD1 (Nucleotide Binding Oligomerization Domain Containing 1), and CGAS (Cyclic GMP-AMP Synthase)) and genes known to interact with SARS-CoV-2 (ADAM9 (ADAM Metallopeptidase Domain 9), FBLN5 (Fibulin 5), FAM8A1 (Family With Sequence Similarity 8 Member A1), CLIP4 (CAP-Gly Domain Containing Linker Protein Family Member 4)))	[27]
Multi-omic rejuvenation of human cells by maturation phase transient reprogramming	Focus on a possible rejuvenation strategy Used Fleischer et al.’s dataset [19] as a reference dataset to train a transcription age-predictor Authors mention collagen I and IV as well as KRT8, KRT18, and APBA2 (Amyloid Beta Precursor Protein Binding Family A Member 2) as downregulated with age and upregulated using their rejuvenation method, and MAF (MAF BZIP Transcription Factor) as being upregulated with age and downregulated upon their rejuvenation method Other genes apparently linking epigenetic and transcriptomic rejuvenation (including FBN2 (Fibrillin 2), TNXB (Tenascin XB), SPTB (Spectrin Beta, Erythrocytic), WISP2 (WNT1-Inducible-Signaling Pathway Protein 2), STRA6 (Signaling Receptor And Transporter Of Retinol STRA6), ASPA (Aspartoacylase))	[28]
BiT age: A transcriptome-based aging clock near the theoretical limit of accuracy	Use of binarized transcriptomic data to establish an accurate age predictor Used *C. elegans* as a model organism and Fleischer et al.’s dataset [19] as a reference dataset to test their model Found that aging-related pathways, such as insulin signaling, are evolutionarily conserved and relevant for multiple species Found enrichment of aging-related genes in their predictor gene set derived from *C. elegans* but did not explicitly analyze these genes instead the authors focused on predicting the biological age correctly	[29]
Genome-wide quantification of ADAR adenosine-to-inosine RNA editing activity	Interested in the quantification of adenosine deaminase that acts on RNA (ADAR) activity Developed the computational tool Alu editing index (AEI) Used Fleischer et al.’s dataset [19] as a reference dataset	[30]
mitoXplorer, a visual data mining platform to systematically analyze and visualize mitochondrial expression dynamics and mutations	Developed the computational tool for quantifying mitochondrial expression dynamics Used Fleischer et al.’s dataset [19] as a reference dataset The authors were interested in genes with mitochondrial function and created a visual data mining platform Focus on Trisomy 21 cells to test their tool	[31]
An integrated pipeline for mammalian genetic screening	Created a pipeline to integrate computational and experimental methods to identify, construct, and induce key regulatory factors Used Fleischer et al.’s dataset [19] as a reference dataset for benchmarking aging genes Authors focused on an integrated solution for systematic mammalian genetic screening studies; no aging-related genes or pathways were analyzed or mentioned	[32]
Landscape of adenosine-to-inosine RNA recoding across human tissues	Generated a highly accurate atlas of A-to-I RNA editing sites within protein-coding regions and their editing levels across human tissues Used Fleischer et al.’s dataset [19] as a reference dataset For regions within the three genes ASNS (Asparagine Synthetase (Glutamine-Hydrolyzing)), NEIL1 (Nei Like DNA Glycosylase 1), and SEMA5B (Semaphorin 5B), the authors calculated multi-species dsRNA structures	[33]
Predicting age from the transcriptome of human dermal fibroblasts	The original study, the authors generated the extensive dataset Used RNA-Seq data to generate a machine learning algorithm for predicting age using the transcriptome	[19]

**Table 2:** Cross check of the genes regarding the common genes in aging and progeria in our RNA-Seq analysis and aging-related/progeria-related proteomics publications.

	Gene Name	Description	Reported Tissue Proteomics	Ref.
Aging Proteomics 1	IGFBP2	insulin like growth factor binding protein 2	Plasma, monocytes, macrophages and precursors	[90,93,94,95]
	STAT1	signal transducer and activator of transcription 1	Plasma, liver	[90,93,96,97]
	TFPI	tissue factor pathway inhibitor	Plasma	[90,94,96]
	KRT18	keratin 18	Plasma, liver	[90,96,97,98]
	CCL2	C-C motif chemokine ligand 2	Plasma	[90,99,100,101]
	IGF1	insulin like growth factor 1	Plasma, cerebrospinal fluid	[90,96,102]
	HGF	hepatocyte growth factor	Plasma, cerebrospinal fluid	[90,93,99,100,102,103]
	MSR1	macrophage scavenger receptor 1	Plasma	[90,93,96]
	EFEMP1	EGF containing fibulin extracellular matrix protein 1	Plasma, urine	[90,93,94,104,105]
	GDF5	growth differentiation factor 5	Plasma, cerebrospinal fluid	[90,96,102]
	KDR	kinase insert domain receptor	Plasma	[90,93,96]
	FST	follistatin	Plasma	[90,93,99]
	SECTM1	secreted and transmembrane 1	Plasma	[90,94,96]
	HS3ST3A1	heparan sulfate-glucosamine 3-sulfotransferase 3A1	Plasma	[90,93,96]
	SPINT2	serine peptidase inhibitor, Kunitz type 2	Plasma, cerebrospinal fluid	[90,93,96,106]
Aging Proteomics 2	IGFBP2	insulin like growth factor binding protein 2	Plasma, monocytes, macrophages and precursors	[90,91,93,94,95]
	STAT1	signal transducer and activator of transcription 1	Plasma, liver	[90,91,93,96,97]
	TFPI	tissue factor pathway inhibitor	Plasma	[90,91,94,96]
Proteomics Fibroblasts	Wnt5A	Wnt family member 5A	Fibroblasts	[92]

**Table 3:** Summary of the DEGs of special interest in the different proteomics studies. Columns indicate if/how often the gene was mentioned in aging-related proteomics studies (according to Johnson et al., 2020), Tendency in gene regulation observed in the RNA-Seq data analysis by Mateos et al. (2018), the iTRAQ analyses of Mateos et al. (2018), in our study regarding aging, in our study regarding progeria.

Gene	Observed in … Proteomics Studies (Johnson et al.)	Tendency Observed in RNA-Seq Progeria (Mateos et al.)	Tendency Observed in iTRAQ Progeria (Mateos et al.)	Tendency Observed in Our Study (Aging)	Tendency Observed in Our Study (Progeria)
IGFBP2	3	up	up	up	up
IGF1	2	down	-	down	down
WNT16	-	up	-	up	up
UCP2	-	up	-	down	up
ACKR4	-	-	-	up	down
CCL2	2	up	-	up	up
KRT8	-	up	-	down	up
KRT18	3	up	-	down	up
ADAMTS15	-	up	-	down	up
ACTN4P1	-	-	-	down	up

## Data Availability

Not applicable.

## Supplementary Materials

The following supporting information can be downloaded at: https://www.mdpi.com/article/10.3390/biomedicines10102440/s1, Figure S1: CNET Plot of Enriched Hallmark Pathways when Comparing RNA Sequencing Data of HGPS Patients and Healthy Children; Figure S2: CNET Plot of Enriched Biological Processes when Comparing RNA Sequencing Data of HGPS Patients and Healthy Children; Figure S3: CNET Plot of Enriched Hallmark Pathways when Comparing RNA Sequencing Data of Nonagenarians and Healthy Children; Figure S4: CNET Plot of Enriched Biological Processes when Comparing RNA Sequencing Data of Nonagenarians and Healthy Children; Figure S5: Visualization of the DEGs involved in ECP using the log2foldchange Data of Comparing Nonagenarians and Healthy Children; Figure S6: miRNA Prediction and Network Visualization of miRNAs Related to WNT16 when Comparing Progeria Patients and Healthy Children; Figure S7: miRNA Prediction and Network Visualization of miRNAs Related to IGFBP2 when Comparing Progeria Patients and Healthy Children; Figure S8: miRNA Prediction and Network Visualization of miRNAs Related to UCP2 when Comparing Progeria Patients and Healthy Children; Table S1: Sample Information; Table S2: Overview Up- and Downregulated DEGs; Table S3: Gene Enrichment Biological Processes Analyses; Table S4: Predicted miRNAs; Table S5: Progeria DEGs (comparison progeria patients and healthy children); Table S6: Aging DEGs (comparison nonagenarians and healthy children); Document S1: Mini-Tutorial [34,36,37,38,39,40,41,42,43,44,46,47,48,49,50,51,57,58,59,62,63,64,158,159,160,161,162].

## Abbrevations

CC	Cellular component
CD4+ T cells	T helper cells also known as CD4-positive cells (CD4 = cluster of differentiation 4)
DEGs	differentially expressed genes
E2F	group of genes encoding transcription factors in higher eukaryotes
ECP	Epithelial Cell Proliferation
FDA	U.S. Food and Drug Administration
FTIs	farnesyltransferase inhibitors
G2M checkpoint	G2/M checkpoint
GEO	Gene Expression Omnibus
GO	Gene Ontology
GO BPs	Gene Ontology enriched biological processes
GSEA	Gene Set Enrichment Analysis
HGPS	Hutchinson-Gilford progeria syndrome
hsa-mir-	human microRNA
iTRAQ	isobaric tag for relative and absolute quantification
KEGG	Kyoto Encyclopedia of Genes and Genomes
MF	molecular function
miR-	microRNA
miRNAs	microRNAs
miRs	microRNAs
MSigDB	Molecular Signatures Database
NES	normalized enrichment score
PCA	Principal component analysis
PRF	Progeria Research Foundation
RNA	ribonucleic acid
RNA-Seq	RNA sequencing
STRING	Search Tool for Retrieval of Interacting Genes/Proteins
UV	ultraviolet
UVB	type B ultraviolet
**Genes**	
ACE2	Angiotensin-converting enzyme 2
ACKR2	Atypical chemokine receptor 2
ACKR4	Atypical chemokine receptor 4
ACTN4P1	Actinin alpha 4 pseudogene 1
ADAM9	ADAM Metallopeptidase Domain 9
ADAMTS	A disintegrin and metalloproteinase with thrombospondin motifs
ADAMTS15	ADAM metallopeptidase with thrombospondin type 1 motif 15
ANLN	Anillin, Actin Binding Protein
APBA2	Amyloid Beta Precursor Protein Binding Family A Member 2
ASNS	Asparagine Synthetase (Glutamine-Hydrolyzing)
ASPA	Aspartoacylase
CCL2	C-C Motif Chemokine Ligand 2
CCL26	C-C Motif Chemokine Ligand 26
CCR10	C-C chemokine receptor type 10
CCR11	Abbreviation for Atypical chemokine receptor 4 (ACKR4)
CDH1	Cadherin 1
CDK1	Cyclin Dependent Kinase 1
CDK4	Cyclin Dependent Kinase 4
CDKN2B	Cyclin Dependent Kinase Inhibitor 2B
CFH	Complement Factor H
CGAS	Cyclic GMP-AMP Synthase
CITED2	Cbp/P300 Interacting Transactivator With Glu/Asp Rich Carboxy-Terminal Domain 2
cKRT18	caspase-cleaved fragment of keratin 18 (KRT18)
CLIP4	CAP-Gly Domain Containing Linker Protein Family Member 4
CPNE1	Copine 1
DLGAP5	DLG Associated Protein 5
DTYMK	Deoxythymidylate Kinase
ECM2	Extracellular Matrix Protein 2
EDIL3	EGF Like Repeats And Discoidin Domains 3
EFEMP1	EGF containing fibulin extracellular matrix protein 1
EGR1	Early Growth Response 1
FAM8A1	Family With Sequence Similarity 8 Member A1
FBN2	Fibrillin 2
FBLN5	Fibulin 5
FST	Follistatin
GDF5	Growth Differentiation Factor 5
HGF	Hepatocyte Growth Factor
HS3ST3A1	Heparan Sulfate-Glucosamine 3-Sulfotransferase 3A1
IGF1	insulin-like growth factor 1
IGFBP1	Insulin Like Growth Factor Binding Protein 1
IGFBP2	Insulin Like Growth Factor Binding Protein 2
IGFBP7	Insulin Like Growth Factor Binding Protein 7
IL11	Interleukin 11
IL13RA2	Interleukin 13 Receptor Subunit Alpha 2
IRF6	Interferon Regulatory Factor 6
KDR	Kinase Insert Domain Receptor
KIFC1	Kinesin Family Member C1
KIT	KIT Proto-Oncogene, Receptor Tyrosine Kinase
KLHL24	Kelch Like Family Member 24
KRAS	Kristen rat sarcoma virus
KRT18	Keratin 18
KRT8	Keratin 8
LEPR	Leptin Receptor
LMNA	Lamin A/C
LMNB1	Lamin B1
LMNB2	Lamin B2
MAF	MAF BZIP Transcription Factor
MCP-1	monocyte chemoattractant protein-1
MKI67	Marker Of Proliferation Ki-67
MMP10	Matrix Metallopeptidase 10
MSR1	Macrophage Scavenger Receptor 1
MYL9	Myosin Light Chain 9
NEIL1	Nei Like DNA Glycosylase 1
NTN4	Netrin 4
NKX3-1	NK3 Homeobox 1
NOD1	Nucleotide Binding Oligomerization Domain Containing 1
PLSCR4	Phospholipid Scramblase 4
POLR2F	RNA Polymerase II, I And III Subunit F
POSTN	Periostin
PRPS1	Phosphoribosyl Pyrophosphate Synthetase 1
PTPRN	Protein Tyrosine Phosphatase Receptor Type N
SECTM1	Secreted and Transmembrane 1
SEMA3D	Semaphorin 3D
SEMA5B	Semaphorin 5B
SIX1	SIX Homeobox 1/Sine Oculis Homeobox Homolog 1
SNAI1	Snail Family Transcriptional Repressor 1
SNAP23	Synaptosome Associated Protein 23
SPINT2	Serine Peptidase Inhibitor, Kunitz Type 2
SPTB	Spectrin Beta, Erythrocytic
STAT1	Signal Transducer And Activator Of Transcription 1
STAT4	Signal Transducer And Activator Of Transcription 4
STRA6	Signaling Receptor And Transporter Of Retinol STRA6
SVEP1	Sushi, Von Willebrand Factor Type A, EGF And Pentraxin Domain Containing 1
TACC3	Transforming Acidic Coiled-Coil Containing Protein 3
TLR3	Toll Like Receptor 3
TLR4	Toll Like Receptor 4
TNXB	Tenascin XB
TOR1AIP1	Torsin 1A Interacting Protein 1 (also known as LAP1B)
U2AF1	U2 Small Nuclear RNA Auxiliary Factor 1
UBE2D1	Ubiquitin Conjugating Enzyme E2 D1
UCP2	Uncoupling Protein 2
WISP2	WNT1-Inducible-Signaling Pathway Protein 2also known as CCN5 (Cellular Communication Network Factor 5)
Wnt	Wingless/Integrated
WNT10B	Wnt Family Member 10B
WNT16	Wnt Family Member 16
WNT5A	Wnt Family Member 5A
