# Supplementary material for: Progeria and Aging—Omics Based Comparative Analysis
Source: Biomedicines. 2022 Sep 29;10(10):2440. doi: 10.3390/biomedicines10102440 (PMC9599154; doi:10.3390/biomedicines10102440)
Supplement: Supplementary file 1 [file biomedicines-10-02440-s001.zip › MiniTutorial.pdf]

Detailed Workflow

As the graphical abstract indicates, the analysis workflow applied in this study consists of several different analysis tools and methods.

In the following, we would like to give a more detailed overview of the methods and packages we used. We will describe how to find and install the relevant packages for researchers interested in bioinformatics but new to the subject. In our study, we used a combination of standard features of the tools described below. Usually, the authors of specific tools also explain how to use their programs/packages in great detail. Since other research projects might also need different features, we decided to link the complete instructions of the respective tools rather than just describing which features of the tools we used.

1.) Data download and Quality Check

Publicly available datasets in the GEO database link to the SRA Run Selector, where the metadata of all samples of the respective set is organized as a table, offering the user the opportunity to select specific samples and download their metadata. The download of the RNA seq data from the Sequence Read Archive [1] can be done in a Linux environment using the SRA Toolkit. The download of the RNA seq data from the Sequence Read Archive [1] can be done in a Linux environment using the SRA Toolkit.

The SRA Toolkit can easily be installed using Miniconda, a software for package management and environment management, which is available here: https://docs.conda.io/en/latest/miniconda.html

To easily install the necessary programs via conda, it is recommended to add these three conda channels:

```
conda config --add channels defaults

conda config --add channels bioconda

conda config --add channels conda-forge
```

After installing the SRA Toolkit (https://anaconda.org/bioconda/sra-tools), the RNA seq data can be downloaded using prefetch and the SRR code of the sample of interest:

```
prefetch SRRxxxxxxx
```

This will download the *.sra file containing the sample's RNA seq data.

Afterward, the *.sra file can be converted into a *.fasta file, which can also be zipped to save space:

fastq-dump -Z --defline-qual '+' SRRxxxxxxx.sra | gzip -c > FILENAME.fastq.gz

FastQC [2] and MultiQC [3] can be used for quality control. FastQC, which is available here https://www.bioinformatics.babraham.ac.uk/projects/fastqc/, gives out single files containing the results of the quality check for each sample. Therefore, subsequently using MultiQC (https://multiqc.info/), which bundles these FastQC-result files into a single file containing all the information, can save time.

If FastQC cannot read a file, there might have been an interruption during the download, and redownloading the file might fix the issue.

2.) Data preparation

In this study, we used the RNA-seq aligner STAR [4] (https://github.com/alexdobin/STAR and https://anaconda.org/bioconda/star) to align the RNA seq data to a reference genome. However, there are several alternatives that require less computational power, for instance, Salmon [5] (https://combine-lab.github.io/salmon/).

As a reference genome, we used the reference genome for human available at GENCODE [6] (https://www.gencodegenes.org/human/): the comprehensive gene annotation on the primary assembly (PRI) as GTF file and the nucleotide sequence of the GRCh38 primary genome assembly (chromosomes and scaffolds, PRI) as fasta file.

Subsequently, we used RSEM [7] to quantify the transcripts. The software is available via conda: https://anaconda.org/bioconda/rsem or the original page https://deweylab.github.io/RSEM/

3.) Data Analysis

The subsequent data analyses used in this study were conducted using R (https://www.r-project.org/) and RStudio (https://www.rstudio.com/products/rstudio/download/) [8]. The required packages can be installed directly via R or using Bioconductor (https://www.bioconductor.org/install/).

DESeq2 [9] is an R package and is available via Bioconductor: https://bioconductor.org/packages/release/bioc/html/DESeq2.html

The DESeq2 "user manual", the vignette is available within R Studio and online at https://bioconductor.org/packages/release/bioc/vignettes/DESeq2/inst/doc/DESeq2.html

In the online version, every analysis step is explained by the authors of the package, and other packages that might be required for analysis, such as tximport [10] (https://bioconductor.org/packages/release/bioc/html/tximport.html) or apeglm [11] (https://www.bioconductor.org/packages/release/bioc/html/apeglm.html) are linked.

4.) Data Visualization

To visualize changes in gene expression, we used a feature (switchPlotGeneExp) of the IsoformSwitchAnalyzeR package [12,13]. The package is also available via Bioconductor (https://www.bioconductor.org/packages/release/bioc/html/IsoformSwitchAnalyzeR.html) and is described in detail at https://bioconductor.org/packages/devel/bioc/vignettes/IsoformSwitchAnalyzeR/inst/doc/IsoformSwitchAnalyzeR.html

Heatmaps were generated using the pheatmap package [14] (https://github.com/raivokolde/pheatmap), which is available via CRAN (https://cran.r-project.org/web/packages/pheatmap/index.html).

Volcano plots were generated using EnhancedVolcano [15] (https://bioconductor.org/packages/release/bioc/html/EnhancedVolcano.html), which also offers a detailed online manual (https://bioconductor.org/packages/release/bioc/vignettes/EnhancedVolcano/inst/doc/EnhancedVolcano.html).

5.) Pathway Enrichment Analysis

The R package clusterProfiler [16] (https://bioconductor.org/packages/release/bioc/html/clusterProfiler.html and https://bioconductor.org/packages/devel/bioc/vignettes/clusterProfiler/inst/doc/clusterProfiler.html), which was used for pathway enrichment analysis in combination with the Molecular Signatures Database [17-20] (https://www.gsea-msigdb.org/gsea/msigdb/), even offers a whole book as a vignette: https://yulab-smu.top/biomedical-knowledge-mining-book/index.html

6.) Protein-protein Interactions

Protein-protein Interactions can be analyzed online at https://string-db.org/ using the STRING database [21,22].

The website offers a detailed manual (https://string-db.org/cgi/help?sessionId=bYTPO2bbRjel) and several tutorial videos (https://string-db.org/help//videos/) explaining how to use the different features and how to use the resulting data with other tools such as Cytoscape [23].

7.) Cytoscape

Cytoscape [23] (https://cytoscape.org/), which offers numerous tutorials at https://github.com/cytoscape/cytoscape-tutorials/wiki, allows the visualization of networks, including STRING and miRNet analyses, and many other functions.

It can be installed on Windows, Mac OS and Linux and does not require previous bioinformatics training, as there are also detailed protocols for biologists without bioinformatics knowledge available, such as the protocol "Pathway enrichment analysis and visualization of omics data using g:Profiler, GSEA, Cytoscape and EnrichmentMap" by Reimand et al. [24].

Cytoscape is not only a tool for visualization on its own but can be enhanced with several Cytoscape applications adding more functionalities to Cyctoscape. For instance, there are various applications for enrichment map visualization, which can be installed on their own or as a collection [24]. These applications are conveniently available via the Cytoscape App Store (https://apps.cytoscape.org/), which makes Cytoscape extremely user-friendly.

8.) miRNA prediction

Predicting miRNAs was also done online via miRNet [25] (https://www.mirnet.ca/). For our study, we used the "Genes" module but there are several other analysis options available. How to use miRNet and its various features is described in several tutorials: https://www.mirnet.ca/miRNet/docs/Tutorial.xhtml

9.) Finding Ligand-Receptor-Interactions Based on Prior Knowledge

The last analysis method we introduced in this article is NicheNet [26] (https://rdrr.io/github/browaeysrobin/nichenetr/), which is described in detail in several tutorials available at the respective GitHub site: https://github.com/saeyslab/nichenetr

Besides using the original database, which is available via zenodo (https://zenodo.org/record/3260758#.YrcLXHZByMo), it is also possible to implement other databases such as the Omnipath database [27] (https://omnipathdb.org/), using a workflow which is described in detail at https://workflows.omnipathdb.org/nichenet1.html

References:

1. Leinonen, R.; Sugawara, H.; Shumway, M.; on behalf of the International Nucleotide Sequence Database, C. The Sequence Read Archive. *Nucleic Acids Research* **2011**, *39*, D19-D21, doi:10.1093/nar/gkq1019.
2. Andrews, S. FastQC: A Quality Control Tool for High Throughput Sequence Data [Online]. Available online at: http://www.bioinformatics.babraham.ac.uk/projects/fastqc/. **2010**.

3. Ewels, P.; Magnusson, M.; Lundin, S.; Käller, M. MultiQC: summarize analysis results for multiple tools and samples in a single report. *Bioinformatics* **2016**, *32*, 3047-3048, doi:10.1093/bioinformatics/btw354.
4. Dobin, A.; Davis, C.A.; Schlesinger, F.; Drenkow, J.; Zaleski, C.; Jha, S.; Batut, P.; Chaisson, M.; Gingeras, T.R. STAR: ultrafast universal RNA-seq aligner. *Bioinformatics* **2013**, *29*, 15-21, doi:10.1093/bioinformatics/bts635.
5. Patro, R.; Duggal, G.; Love, M.I.; Irizarry, R.A.; Kingsford, C. Salmon provides fast and bias-aware quantification of transcript expression. *Nature Methods* **2017**, *14*, 417-419, doi:10.1038/nmeth.4197.
6. Frankish, A.; Diekhans, M.; Ferreira, A.-M.; Johnson, R.; Jungreis, I.; Loveland, J.; Mudge, J.M.; Sisu, C.; Wright, J.; Armstrong, J.; et al. GENCODE reference annotation for the human and mouse genomes. *Nucleic Acids Research* **2019**, *47*, D766-D773, doi:10.1093/nar/gky955.
7. Li, B.; Dewey, C.N. RSEM: accurate transcript quantification from RNA-Seq data with or without a reference genome. *BMC Bioinformatics* **2011**, *12*, 323, doi:10.1186/1471-2105-12-323.
8. R Core Team. R: A Language and Environment for Statistical Computing. **2021**.
9. Love, M.I.; Huber, W.; Anders, S. Moderated estimation of fold change and dispersion for RNA-seq data with DESeq2. *Genome Biology* **2014**, *15*, 550, doi:10.1186/s13059-014-0550-8.
10. Soneson, C.; Love, M.; Robinson, M. Differential analyses for RNA-seq: transcript-level estimates improve gene-level inferences [version 2; peer review: 2 approved]. *F1000Research* **2016**, *4*, doi:10.12688/f1000research.7563.2.
11. Zhu, A.; Ibrahim, J.G.; Love, M.I. Heavy-tailed prior distributions for sequence count data: removing the noise and preserving large differences. *Bioinformatics* **2019**, *35*, 2084-2092, doi:10.1093/bioinformatics/bty895.
12. Vitting-Seerup, K.; Sandelin, A. The Landscape of Isoform Switches in Human Cancers. *Molecular Cancer Research* **2017**, *15*, 1206-1220, doi:10.1158/1541-7786.MCR-16-0459.
13. Vitting-Seerup, K.; Sandelin, A. IsoformSwitchAnalyzeR: analysis of changes in genome-wide patterns of alternative splicing and its functional consequences. *Bioinformatics* **2019**, *35*, 4469-4471, doi:10.1093/bioinformatics/btz247.
14. Kolde, R. pheatmap: Pretty Heatmaps. R package version 1.0.12. https://CRAN.R-project.org/package=pheatmap. **2019**.
15. Blighe, K.; Rana, S.; Lewis, M. EnhancedVolcano: Publication-ready volcano plots with enhanced colouring and labeling. **2022**.
16. Yu, G.; Wang, L.-G.; Han, Y.; He, Q.-Y. clusterProfiler: an R Package for Comparing Biological Themes Among Gene Clusters. *OMICS: A Journal of Integrative Biology* **2012**, *16*, 284-287, doi:10.1089/omi.2011.0118.
17. Subramanian, A.; Tamayo, P.; Mootha Vamsi, K.; Mukherjee, S.; Ebert Benjamin, L.; Gillette Michael, A.; Paulovich, A.; Pomeroy Scott, L.; Golub Todd, R.; Lander Eric, S.; et al. Gene set enrichment analysis: A knowledge-based approach for interpreting genome-wide expression profiles. *Proceedings of the National Academy of Sciences* **2005**, *102*, 15545-15550, doi:10.1073/pnas.0506580102.
18. Liberzon, A.; Birger, C.; Thorvaldsdóttir, H.; Ghandi, M.; Mesirov, J.P.; Tamayo, P. The Molecular Signatures Database (MSigDB) hallmark gene set collection. *Cell systems* **2015**, *1*, 417-425, doi:10.1016/j.cels.2015.12.004.

19. Ashburner, M.; Ball, C.A.; Blake, J.A.; Botstein, D.; Butler, H.; Cherry, J.M.; Davis, A.P.; Dolinski, K.; Dwight, S.S.; Eppig, J.T.; et al. Gene Ontology: tool for the unification of biology. *Nature Genetics* **2000**, *25*, 25-29, doi:10.1038/75556.
20. The Gene Ontology, C. The Gene Ontology resource: enriching a GOld mine. *Nucleic Acids Research* **2021**, *49*, D325-D334, doi:10.1093/nar/gkaa1113.
21. Szklarczyk, D.; Gable, A.L.; Lyon, D.; Junge, A.; Wyder, S.; Huerta-Cepas, J.; Simonovic, M.; Doncheva, N.T.; Morris, J.H.; Bork, P.; et al. STRING v11: protein–protein association networks with increased coverage, supporting functional discovery in genome-wide experimental datasets. *Nucleic Acids Research* **2019**, *47*, D607-D613, doi:10.1093/nar/gky1131.
22. Jensen, L.J.; Kuhn, M.; Stark, M.; Chaffron, S.; Creevey, C.; Muller, J.; Doerks, T.; Julien, P.; Roth, A.; Simonovic, M.; et al. STRING 8—a global view on proteins and their functional interactions in 630 organisms. *Nucleic Acids Research* **2009**, *37*, D412-D416, doi:10.1093/nar/gkn760.
23. Shannon, P.; Markiel, A.; Ozier, O.; Baliga, N.S.; Wang, J.T.; Ramage, D.; Amin, N.; Schwikowski, B.; Ideker, T. Cytoscape: A Software Environment for Integrated Models of Biomolecular Interaction Networks. *Genome Research* **2003**, *13*, 2498-2504, doi:10.1101/gr.1239303.
24. Reimand, J.; Isserlin, R.; Voisin, V.; Kucera, M.; Tannus-Lopes, C.; Rostamianfar, A.; Wadi, L.; Meyer, M.; Wong, J.; Xu, C.; et al. Pathway enrichment analysis and visualization of omics data using g:Profiler, GSEA, Cytoscape and EnrichmentMap. *Nature Protocols* **2019**, *14*, 482-517, doi:10.1038/s41596-018-0103-9.
25. Chang, L.; Zhou, G.; Soufan, O.; Xia, J. miRNet 2.0: network-based visual analytics for miRNA functional analysis and systems biology. *Nucleic Acids Research* **2020**, *48*, W244-W251, doi:10.1093/nar/gkaa467.
26. Browaeys, R.; Saelens, W.; Saeys, Y. NicheNet: modeling intercellular communication by linking ligands to target genes. *Nature Methods* **2020**, *17*, 159-162, doi:10.1038/s41592-019-0667-5.
27. Türei, D.; Valdeolivas, A.; Gul, L.; Palacio-Escat, N.; Klein, M.; Ivanova, O.; Ölbei, M.; Gábor, A.; Theis, F.; Módos, D.; et al. Integrated intra- and intercellular signaling knowledge for multicellular omics analysis. *Molecular Systems Biology* **2021**, *17*, e9923, doi:https://doi.org/10.15252/msb.20209923.
