# Supplementary material for: Progeria and Aging—Omics Based Comparative Analysis
Source: Biomedicines. 2022 Sep 29;10(10):2440. doi: 10.3390/biomedicines10102440 (PMC9599154; doi:10.3390/biomedicines10102440)
Supplement: Supplementary file 1 [file biomedicines-10-02440-s001.zip › SupplementaryFigures_final.pdf]

Supplementary Figures

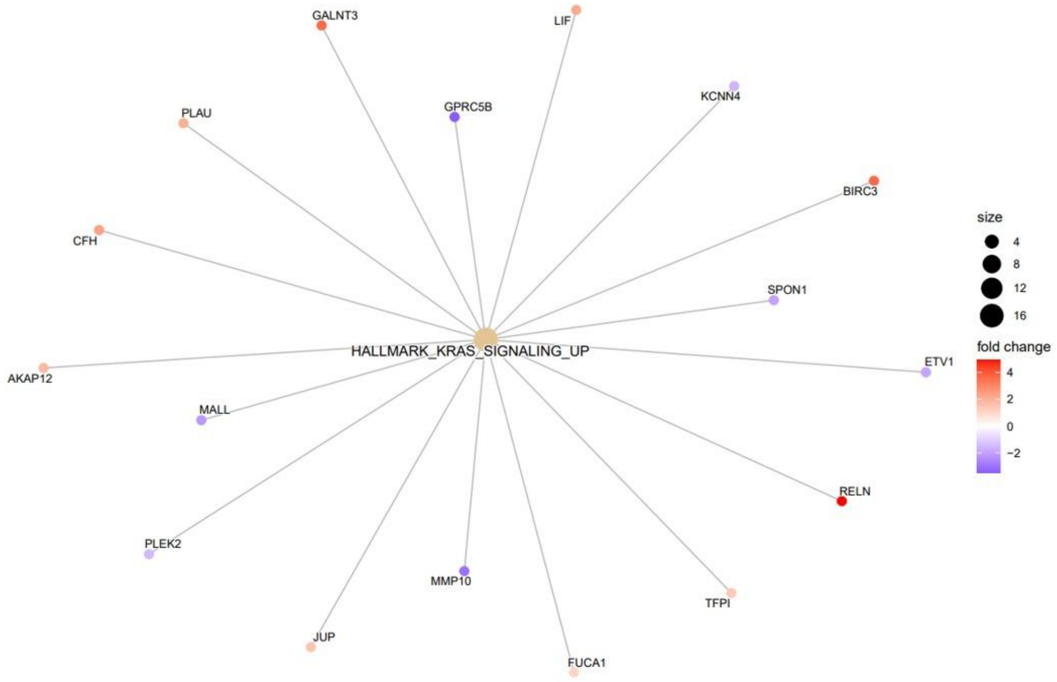

Supplemental Figure S1. Comparing gene expression in RNA sequencing data of HGPS patients and healthy children. CNET plot of enriched hallmark pathways. Red indicates upregulation in HGPS compared to healthy children, blue downregulation (derived from log2fold change according to our DESeq2 analysis, lines indicate predicted interactions. Plot was generated using clusterProfiler (version 4.4.2) with the MSigDB (version 7.5.1) database).

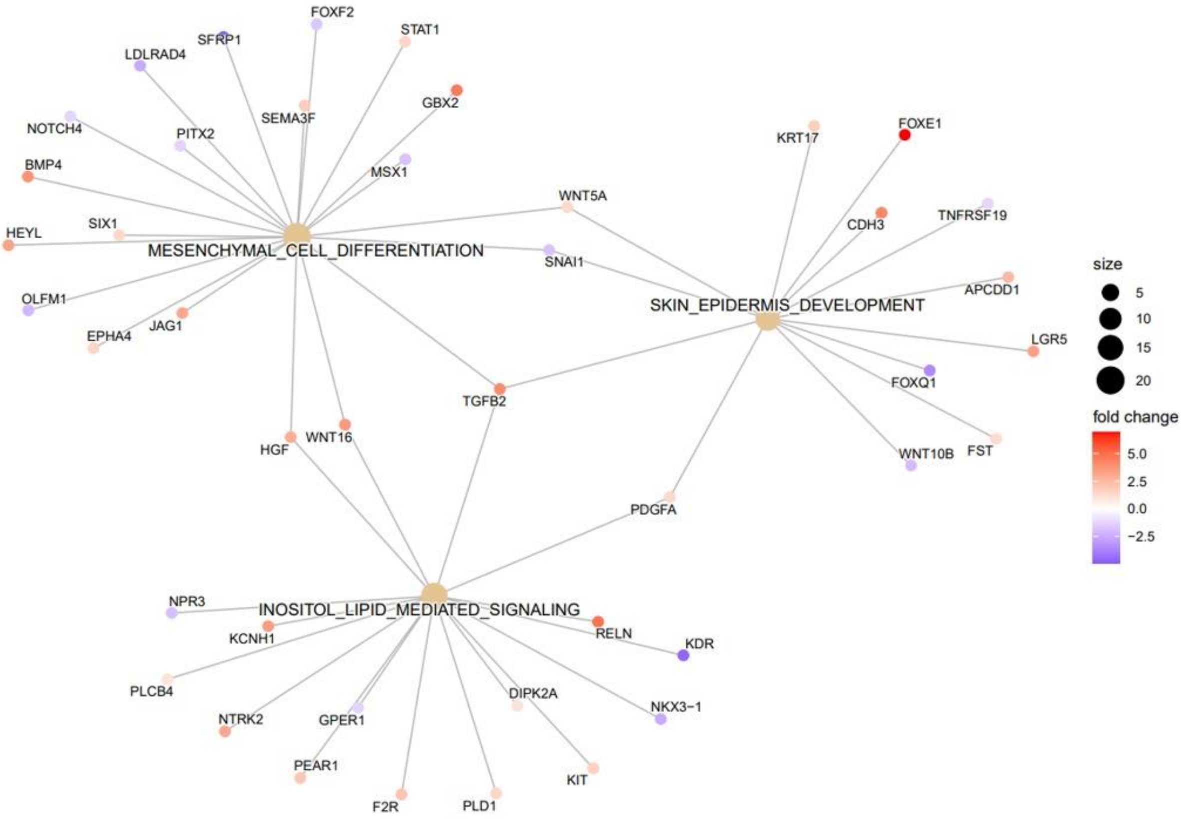

Supplemental Figure S2. Comparing gene expression in RNA sequencing data of HGPS patients and healthy children. CNET plot of GO enriched biological processes. Red indicates upregulation in HGPS compared to healthy children, blue downregulation (derived from log2fold change according to our DESeq2 analysis, lines indicate predicted interactions. Plot was generated using clusterProfiler (version 4.4.2) with the MSigDB (version 7.5.1) database).

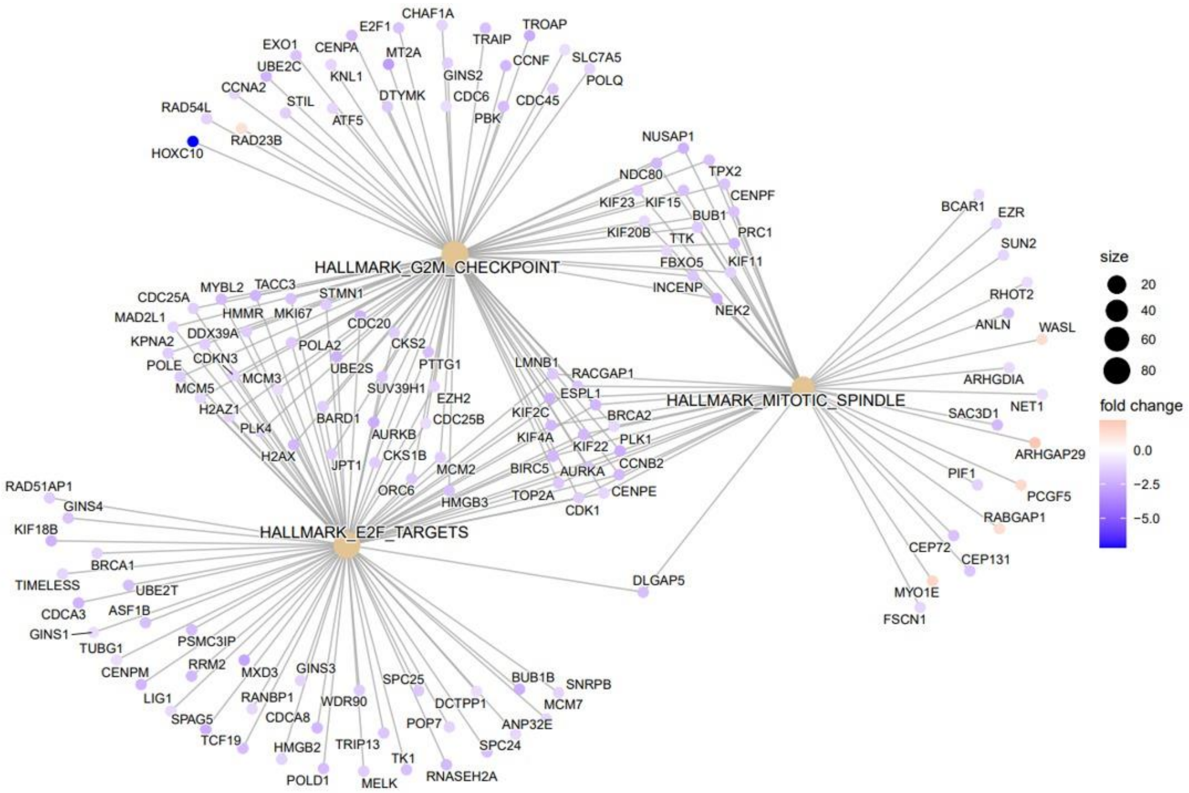

Supplemental Figure S3. Comparing gene expression in RNA sequencing data of nonagenarians and healthy children. CNET plot of enriched hallmark pathways. Red indicates upregulation in nonagenarians compared to healthy children, blue downregulation (derived from log2fold change according to our DESeq2 analysis, lines indicate predicted interactions. Plot was generated using clusterProfiler (version 4.4.2) with the MSigDB (version 7.5.1) database).

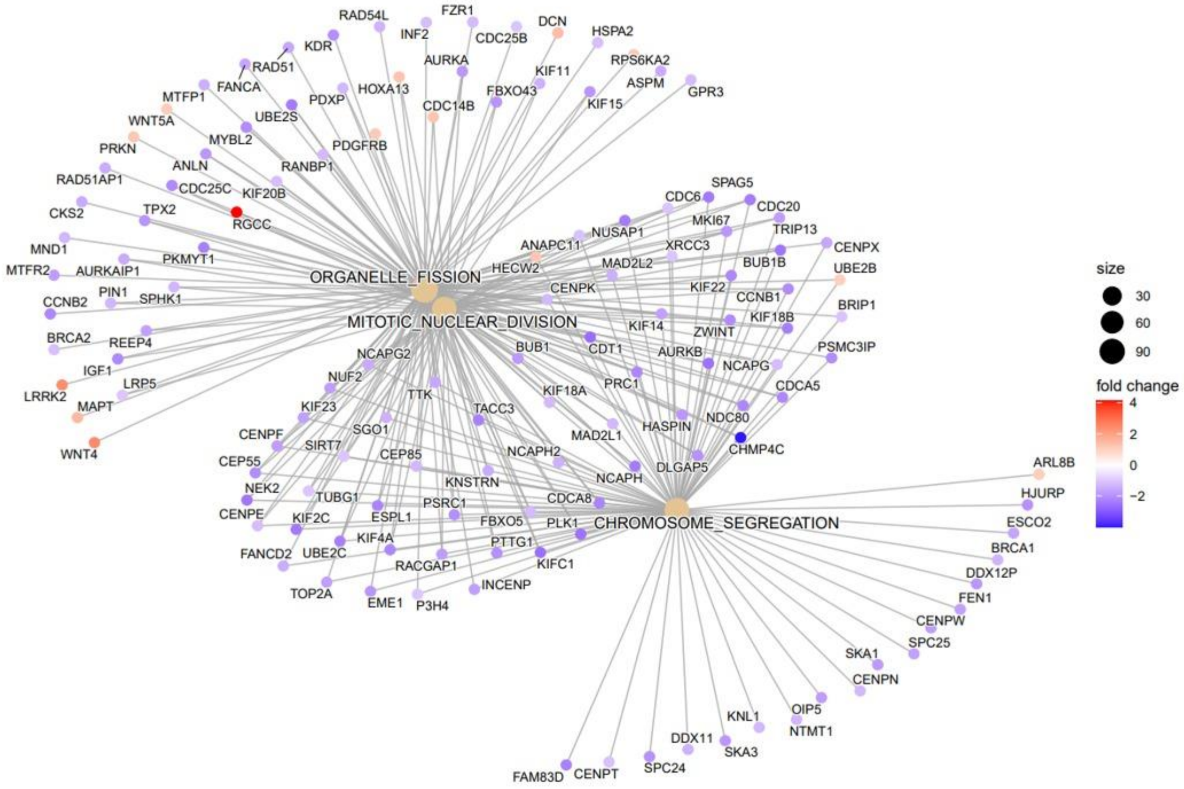

Supplemental Figure S4. Comparing gene expression in RNA sequencing data of nonagenarians and healthy children. CNET plot of GO enriched biological processes. Red indicates upregulation in nonagenarians compared to healthy children, blue downregulation (derived from log2fold change according to our DESeq2 analysis, lines indicate predicted interactions. Plot was generated using clusterProfiler (version 4.4.2) with the MSigDB (version 7.5.1) database).

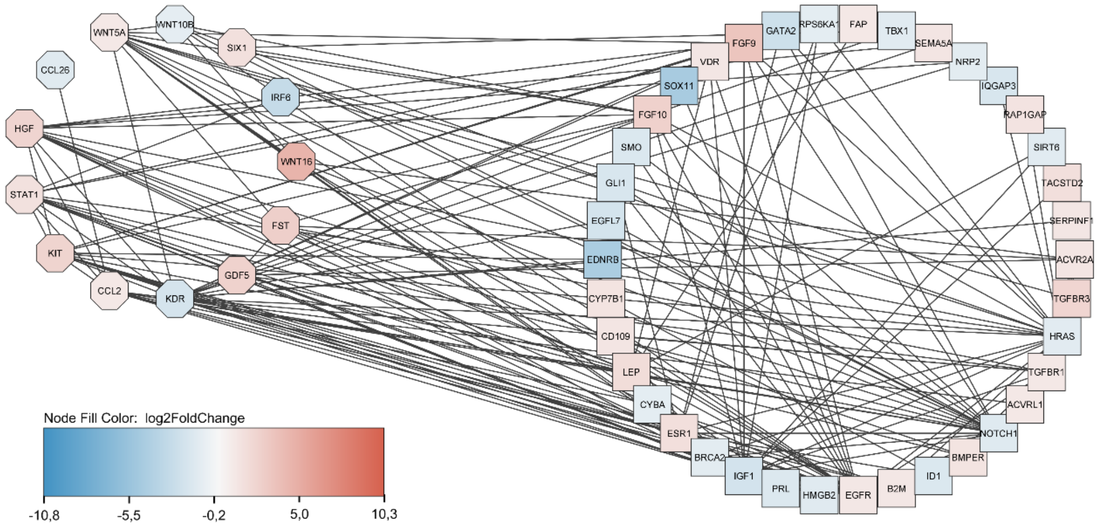

Supplemental Figure S5 Visualization of the DEGs involved in ECP (by using STRING database and Cytoscape): common DEGs (octagons) and the DEGs specific for comparing nonagenarians and healthy children (rectangles) and the respective log2foldchanges (blue downregulated, red upregulated, derived from log2fold change according to our DESeq2 analysis, lines indicate predicted interactions. Plot was generated by importing the STRING database analysis results (applying fullstringnetwork medium confidence of 0.4) to Cytoscape).

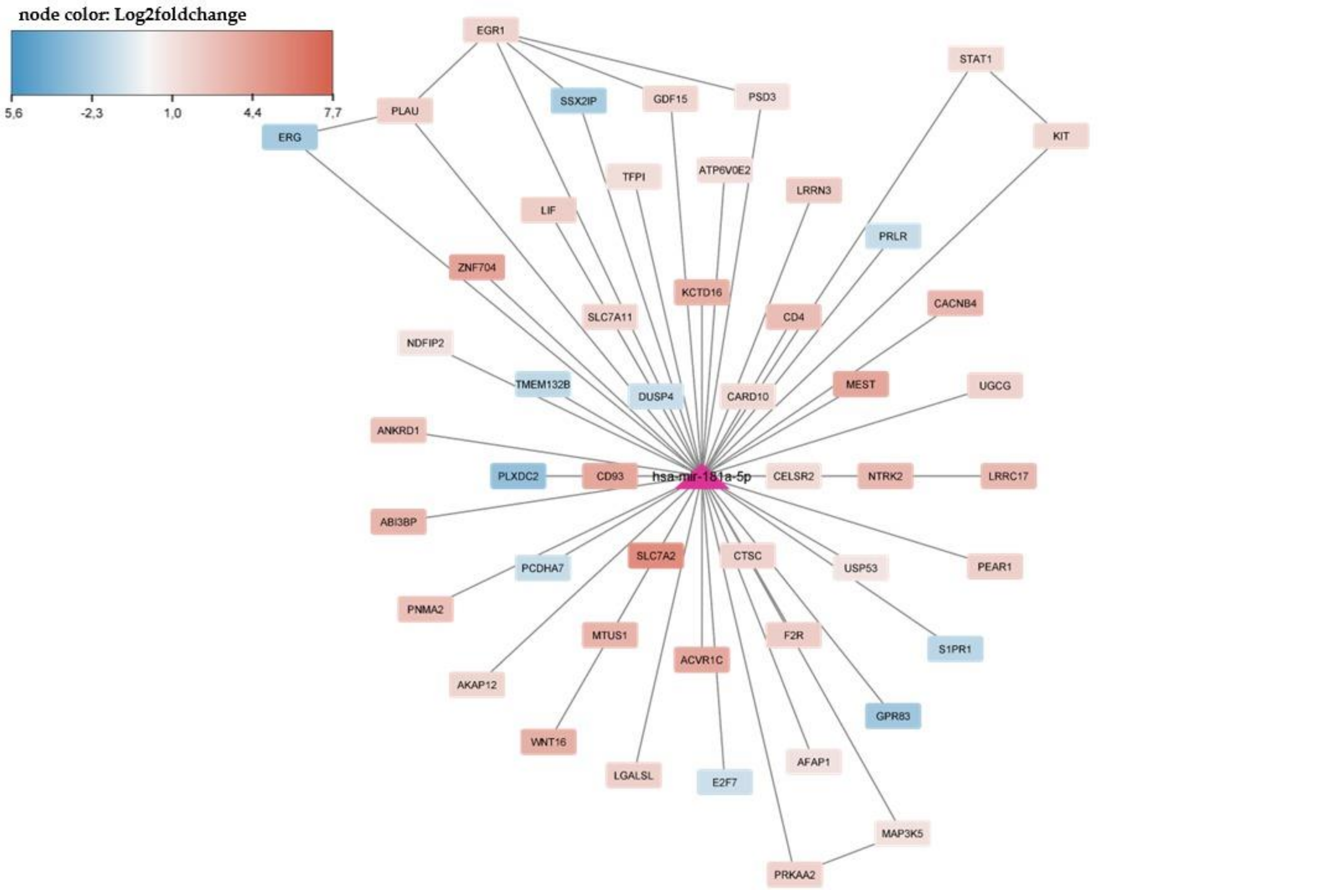

Supplemental Figure S 6. miRNA prediction and network visualization of miRNAs related to WNT16 when comparing progeria patients and healthy children. Red indicates upregulation in HGPS compared to healthy children, blue downregulation (derived from log2fold change according to our DESeq2 analysis, visualization of the miRNet output via Cytoscape, lines indicate predicted interactions).

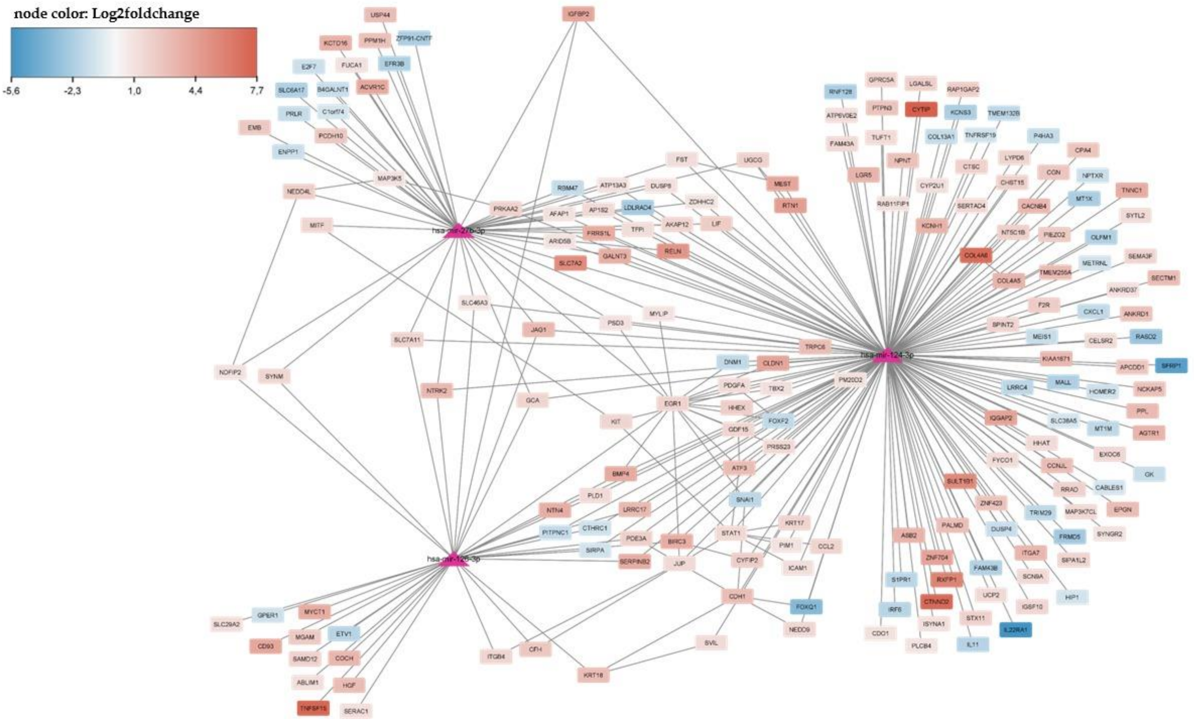

Supplemental Figure S7. miRNA prediction and network visualization of miRNAs related to IGFBP2 when comparing progeria patients and healthy children. Red indicates upregulation in HGPS compared to healthy children, blue downregulation (derived from log2fold change according to our DESeq2 analysis, visualization of the miRNet output via Cytoscape, lines indicate predicted interactions).

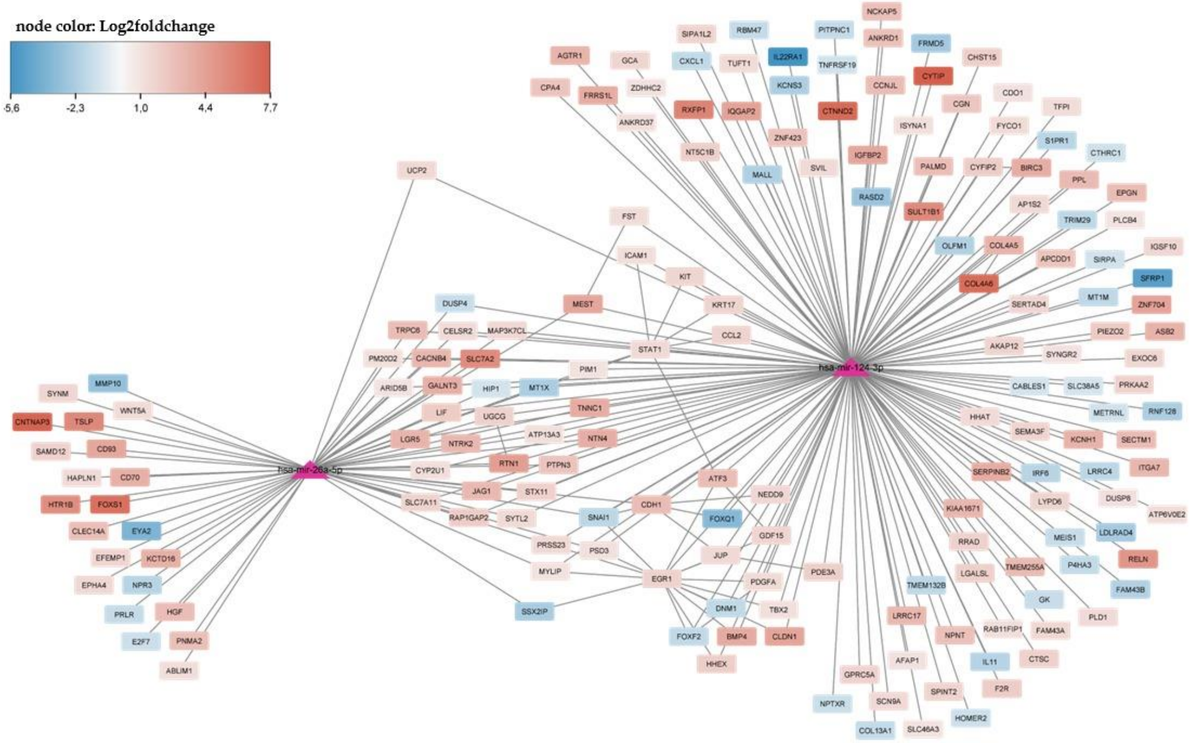

Supplemental Figure S8. miRNA prediction and network visualization of miRNAs related to UCP2 when comparing progeria patients and healthy children. Red indicates upregulation in HGPS compared to healthy children, blue downregulation (derived from log2fold change according to our DESeq2 analysis, visualization of the miRNet output via Cytoscape, lines indicate predicted interactions).
